# Predictive Model Building for Aggregation Kinetics Based on Molecular Dynamics Simulations of an Antibody Fragment

**DOI:** 10.1021/acs.molpharmaceut.4c00859

**Published:** 2024-09-30

**Authors:** Yuhan Wang, Hywel D. Williams, Duygu Dikicioglu, Paul A. Dalby

**Affiliations:** 1 Department of Biochemical Engineering, University College London, London WC1E 6BT, U.K.; 2 Biopharmaceutical Product Development, CSL Ltd., 45 Poplar Road, Parkville 3052, Australia

**Keywords:** antibody aggregation, protein stability, monoclonal antibody, formulation, feature engineering, regression analysis, molecular dynamics

## Abstract

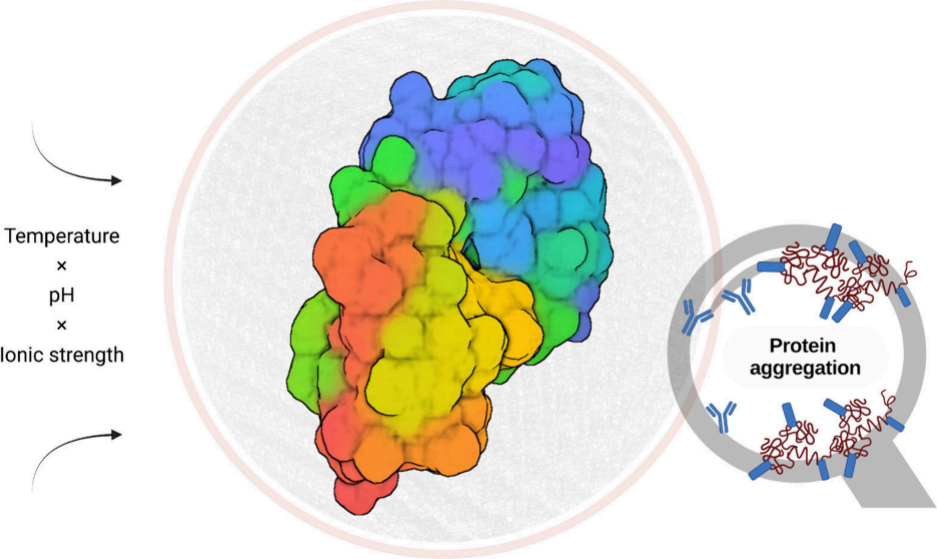

Computational methods including machine learning and molecular dynamics simulations have strong potential to characterize, understand, and ultimately predict the properties of proteins relevant to their stability and function as therapeutics. Such methods would streamline the development pathway by minimizing the current experimental testing required for many protein variants and formulations. The molecular understanding of thermostability and aggregation propensity has advanced significantly along with predictive algorithms based on the sequence-level or structural-level information on a protein. However, these approaches focus largely on a comparison of protein sequence variations to correlate the properties of proteins to their stability, solubility, and aggregation propensity. For therapeutic protein development, it is of equal importance to take into account the impact of the formulation conditions to elucidate and predict the stability of the antibody drugs. At the macroscopic level, changing temperature, pH, ionic strength, and the addition of excipients can significantly alter the kinetics of protein aggregation. The mechanisms controlling aggregation kinetics have been traced back to a combination of molecular features, including conformational stability, partial unfolding to aggregation-prone states, and the colloidal stability governed by surface charges and hydrophobicity. However, very little has been done to evaluate these features in the context of protein dynamics in different formulations. In this work, we have combined a range of molecular features calculated from the Fab A33 protein sequence and molecular dynamics simulations. Using the power of advanced, yet interpretable, statistical tools, it has been possible to uncover greater insights into the mechanisms behind protein stability, validating previous findings, and also develop models that can predict the aggregation kinetics within a range of 49 different solution conditions.

## Introduction

Measuring the physicochemical and biophysical properties of proteins, such as self-interaction and aggregation, thermal stability, and colloidal stability, is a critical route for evaluating the developability and formulation performance of antibody-based therapies prior to process development and real-time formulation testing.^1−8^

Aggregation, the microscopic mechanism of which has not yet been fully understood, is increasingly thought to occur through the partial unfolding of the native protein structure to expose sites that are more prone to self-interaction.^9−19^ Overall, the self-association of proteins is influenced by a combination of conformational changes to the native state, and the repulsive and attractive interactions between proteins leading to larger particles.^2,5,8,20,21^ The surface properties that determine colloidal stability and propensity for self-interaction include a balance and distribution of surface charge, hydrophobicity, and the propensity for cross-β sheet formation. The latter feature is central to specific sequence motifs described as aggregation prone regions (APRs). Conformational changes can include global unfolding in extreme conditions.^22^ However, therapeutic formulations, especially under low temperature storage conditions, will normally only experience local unfolding events^23^ or fluctuations within the native state ensemble.^24^ These changes can alter the overall surface properties of the protein and so also modify the aggregation kinetics, often making the local unfolding events rate limiting.^25^ All of these are modulated by the physical environment provided by the formulation, which can thus alter both the kinetics and the dominant pathways of aggregation.

Many aggregation propensity prediction algorithms have been developed, including sequence-based prediction tools^26−30^ and structure-based prediction tools.^31,31−36^ They offer fast and easy ways to predict protein aggregation at the sequence and, in some cases, at the 3D structure level, without the complexity of handling the higher dimensional data of protein structure movements, which risks ignoring the influence of the protein microenvironment and its impact on dynamics. Several machine learning models have also been developed recently to predict developability behaviors such as aggregation and viscosity from biophysical and physicochemical experimental data sets.^3,37^ These are typically aimed at using developability measures obtained experimentally, often at high throughput, to predict beyond the conditions tested such as at lower temperatures, higher protein concentrations, or over longer time periods. However, their reliance on simple measures may not always be accurately extrapolated to these required conditions and also do not easily enable new formulations to be designed in a predictable manner.

Molecular dynamics simulations, sometimes combined with experimentally obtained biophysical data, have been applied previously to address, understand, or predict protein developability, aggregation, stability, or viscosity in many studies.^12,38−42^ This offers the potential to study protein conformational changes at an atomic level alongside intermolecular interactions by simulating proteins under a wide range of conditions. Kinetics and thermodynamic properties such as aggregation kinetics (ln(*v*)), the fraction of native contacts (*Q*), and thermostability (*T*_m_) are commonly measured experimental parameters that are often compared with molecular dynamics features. *T*_m_ is defined as the temperature at which 50% of a protein is denatured. A study revealed that the fraction of native contacts (*Q*) calculated from MD simulations conducted at 400 K was able to find a good correlation with *T*_m_.^42^

MD simulations have also been used to aid our understanding of protein formulations. For example, MD simulations of three monoclonal antibodies (mAbs) revealed the preferential exclusion of sorbitol, sucrose, or trehalose from the mAb surfaces.^43^ Despite this, significant local interactions occurred, especially with the exposed hydrophobic residues. These interactions, influenced by surface characteristics like hydrophobicity, solvent accessibility, and charge distribution, may shield hydrophobic residues, preventing the unfavorable interactions that lead to aggregation.^44,45^ MD simulations have also explored excipients like mannitol,^46^ sucrose,^46^ hydroxypropyl methylcellulose acetate succinate (HPMCAS),^47^ polyvinylpyrrolidone (PVP),^48^ phosphate,^49^ citrate,^49^ glycerol,^50^ and 2-hydroxypropyl-β-cyclodextrin (HPβCD),^51^ providing mechanistic insights into excipient–protein interactions that might impact aggregation, particularly in freeze-dried systems.^51,52^ A recent study used coarse-grained MD simulations to investigate the impact of 41 different types of excipients on protecting the APR-prone regions of human serum albumin and reported that ethoxylated compounds had the best performance as APR-shielding antiaggregation agents.^53^

Building on all of these applications, MD has significant further potential. In particular, various statistical approaches offer the possibility to use multiple features extracted from MD simulations to build models that predict protein properties. Recently for example, machine learning was used to simulate 312 enzyme variants and then predict their stability-dependent expression levels by combining MD and sequence-based features.^54^ MD simulations and various statistical models have also been used to predict antibody aggregation rates or viscosities for a range of therapeutic antibody variants.^38−40^ However, such approaches have yet to be used to investigate the impact of a wide range of formulation conditions on the protein aggregation kinetics.

In the present study, we used all-atom molecular dynamics simulations to investigate the stability and aggregation mechanism of a Fab region (Fab A33) in 49 formulation environments composed of different temperatures, pHs, and salt concentrations. Fab A33 contains 214 residues on the light chain and 228 residues on the heavy chain. The mechanism of aggregation for this particular Fab is already known to be rate-limited by partial unfolding to a native-like state,^11,12,25,55^ making it highly likely to be dependent on native monomer molecular dynamics. These dynamics have the potential to expose one or more of seven aggregation prone regions (APRs),^11,12^ based on a consensus of predictions from four sequence-based algorithms.^26−28,32,56^ The seven APRs predicted are at residues 31–36, 47–51, 114–118, and 129–139 in the light chain and residues 261–265, 325–329, and 387–402 in the heavy chain. A workflow combining MD simulations, molecular feature calculation, feature selection and combination, and then model building was established in this work to predict experimentally determined protein aggregation kinetics. The main analysis methods included linear regression, multiple linear regression, and partial linear regression. Machine learning regression, building interpretable models, was also used as a further attempt to gain more experience in applying this method. By constructing such a workflow, we aimed to use the power of simulations and regression models to identify key conformations of Fab A33 that contribute to aggregation kinetics and protein unfolding.

## Materials and Methods

### All-Atom Molecular Dynamics Simulations

Molecular dynamic (MD) simulations on the 2.5 Å resolution Fab A33 crystal structure (PDB ID: 7NFA)^57^ were conducted in Gromacs MD software,^58^ version 2019.3. Since pH, temperature, and ionic strength can greatly influence protein conformations and stability, the conditions for MD simulations were varied across a combination of 49 different formulation conditions. These were picked from a possible 105 conditions for which experimental data were available.^11^ Fifteen of the conditions were based on a custom design in the design of experiments (DoE), while the other 34 were chosen for their high experimental aggregation rates. A full list of the combinations can be found in Table 1. This investigated four settings of temperature, 277, 296, 318, and 338 K (4 °C, 23 °C, 45 °C, 65 °C), six different pHs, 3.5, 4.5, 5.5, 7.0, 8.0, 9.0, and nine different ionic strengths, 0 mM, 50 mM, 100 mM, 150 mM, 200 mM, 250 mM, 300 mM, 400 mM, 500 mM sodium chloride. Six replicates under each condition gave a total of 324 MD simulation runs. Thus, the combinations of pH, temperature, and ionic strength were matched to experimentally determined aggregation kinetics (ln(*v*)) and thermal stabilities (*T*_m_) for the same Fab. All simulations were run on the UCL Kathleen cluster.

**Table 1:** 49 Different Formulation Conditions and Their Corresponding Experimental Measurements, Melting Temperature (*T*_m_), and Aggregation Rate (ln(*v*))^11^

number	temperature (K)	ionic strength (mM)	pH	melting temperature *T*_m_ (K)	aggregation rate ln(*v*) (*v* in % day^–1^)
1	277	0	3.5	73.7	–5.95
2	338	100	3.5	62.5	9.01
3	338	200	3.5	60.6	9.64
4	277	50	3.5	64.9	–4.38
5	277	150	3.5	61.8	–5.36
6	338	250	3.5	59.5	9.88
7	338	150	3.5	61.8	9.59
8	338	50	3.5	64.9	9.32
9	318	250	3.5	59.5	1.38
10	296	250	3.5	59.5	–3.81
11	296	250	4.5	73.1	–4.19
12	318	150	4.5	73.3	–3.07
13	338	0	4.5	83.2	0.43
14	338	200	4.5	71.9	7.09
15	338	250	4.5	73.1	6.85
16	318	50	4.5	75.0	–3.20
17	296	50	4.5	75.0	–4.12
18	338	50	4.5	75.0	4.90
19	338	100	4.5	74.0	5.60
20	338	150	4.5	73.3	6.54
21	277	250	4.5	73.1	–3.59
22	296	250	5.5	80.7	–3.76
23	296	100	5.5	81.3	–3.68
24	296	0	5.5	79.6	–2.96
25	296	200	5.5	80.3	–3.70
26	296	150	5.5	80.4	–4.09
27	296	50	5.5	79.7	–3.61
28	318	250	7	77.9	–2.71
29	296	50	7	78.0	–4.65
30	296	250	7	77.9	–6.08
31	338	0	7	78.5	0.98
32	318	150	7	78.6	–2.81
33	338	50	7	78.0	0.98
34	277	250	7	77.9	–4.86
35	296	50	7	78.0	–4.65
36	277	150	7	78.6	–5.32
37	296	150	7	78.6	–4.02
38	338	0	8	77.9	1.62
39	338	500	8	77.1	1.83
40	338	400	8	77.2	2.05
41	277	100	8	77.3	–4.82
42	338	300	8	77.9	2.08
43	338	200	8	77.9	2.18
44	338	200	9	77.4	2.47
45	338	100	9	76.7	2.84
46	338	500	9	76.8	2.80
47	338	400	9	76.8	2.86
48	338	0	9	76.5	2.06
49	338	300	9	76.9	2.83

The workflow of the MD simulations can be summarized as follows: establishment of an environmental box for simulation, solvation, addition of ions, energy minimization, equilibrium, and MD production run. To assist the decision-making process in the selection of the appropriate force field, a benchmark study was performed at the beginning of this study to evaluate the differences and similarities between four different force fields on Fab A33 (OPLS,^59^ Amber 94,^60^ Amber 99SB,^61^ Amber 96^62^). The results showed no significant differences (Figures S1–S3). Although longer simulations might be expected to show divergences, the OPLS force field was chosen primarily to be consistent with the previous studies on Fab A33 and to future-proof studies with more complex formulations.^12^ A cubic box was used for defining the boundaries of the systems, and all of the proteins were separated by 10.0 Å from the box boundaries. Energy minimization of each system was performed using steepest descent to achieve the maximum force less than 1000 kJ/mol/nm, followed by equilibration for 100 ps in the constant-volume ensemble (*NVT*) to stabilize at the specified temperature and 100 ps in the constant-pressure ensemble (*NPT*) to stabilize at atmospheric pressure.

### Feature Calculation

MD trajectories were recorded every 0.1 ns (a total of 1001 frames per MD run). To understand the impact of temperature, ionic strength, and pH on the protein structure in comparative detail, a total of 17 molecular features were calculated in this study, including root-mean-square deviation (RMSD), radius of gyration (*R*_g_), root-mean-square fluctuations (RMSF), the number of hydrogen bonds, the solvent accessible surface area (SASA), salt bridges, native contacts, and net charges. A full list of those molecular features, as well as their descriptions, can be found in the table below (Table 2).

**Table 2:** 17 Molecular Features, Their Categories, Descriptions, and the Software Tools Where the Features Were Calculated^a^

number	name	molecular descriptor code in the models	category	description	software to calculate
1	Total solvent accessible surface area (SASA)	Total SASA	Geometrical	The total accessible areas of the solvent molecule on the surface of the α carbon atoms of the protein	GROMACS
2	Nonpolar solvent accessible surface area	Nonpolar SASA	Geometrical	The accessible areas of the solvent molecule on the surface of the nitrogen (N) and oxygen atom (O)	GROMACS
3–9	Delta solvent accessible surface area (Δ*S*ASA) for each of the 7 APR regions	r_31–36^b^	Geometrical	The ΔSASA within each APR was obtained by calculating the values averaged over the last 50 ns and subtracting the values averaged from the first 20 frames.	GROMACS
		r_47–51^b^			
		r_114–118^b^			
		r_129–139^b^			
		r_261–265^b^			
		r_325–329^b^			
		r_387–402^b^			
10	Sum of the Δ*S*ASA of the 7 APRs	sum_aprsasa	Geometrical	The sum of the delta solvent accessible surface area values for the 7 APR regions	GROMACS
11	Average fraction of native contacts	Average native contact (last 50 ns)	Topological	The fraction of native contacts over time	MDanalysis
12	Root mean square fluctuation (RMSF)	Last 50 ns mean RMSF	Spatial (dynamic properties)	It measures the average deviation of a protein residue over time from a reference position	GROMACS
13	Root mean square deviation (RMSD)	Last 50 ns mean *R*_g_	Spatial (dynamic properties)	It measures how much a certain molecular structure deviates from a reference structure	GROMACS
14	Radius of gyration (*R*_g_)	Last 50 ns mean RMSD	Spatial (dynamic properties)	The radius of gyration measures the compactness of a protein structure.	GROMACS
15	Number of hydrogen bonds	Number of hydrogen bonds in the last 50 ns	Topological	The number of hydrogen bonds over time	GROMACS
16	Net charges	Net charges	Electrostatic	The net charge of the protein at different pHs	PropKa
17	Salt bridges	Salt bridge average	Topological	The average of the salt bridge occurrence in the last 50 ns	MDanalysis

a The molecular features were used as inputs for model building including linear regression, multiple linear regression, partial least squares regression, cross-validation, and machine learning.

b r_*x*–*y*: residue numbers, indicating APRs 1–7.

Various molecular features, including total solvent accessible surface area (SASA), nonpolar solvent accessible surface area, solvent accessible surface area (SASA) of 7 APR regions, number of hydrogen bonds, root-mean-square fluctuation (RMSF), radius of gyration (*R*_g_), and all-atom root-mean-square deviation (RMSD), were directly calculated using Gromacs built-in functions. The total SASA feature excluded hydrogen atoms, while nonpolar SASA represented the carbon atom accessibility to the solvent. The solvent accessible surface area (SASA) of 7 APR regions was calculated as the total solvent-accessible area of the residues identified within each of the seven individual APRs. Each SASA was recalculated as the change (Δ*S*ASA) from the SASA at the 20th frame in the simulation subtracted from the average SASA over the last 50% frames of the trajectory. The ΔSASA for each individual APR region was also summed as the sum of the APR ΔSASA. All-atom root-mean-square deviation (RMSD) values were calculated relative to the first frame structure.

Average fraction of native contacts, and salt bridge occurrences were calculated with the Python library MDanalysis.^63,64^ The native contacts were determined by identifying all α-carbon contacts within the protein, employing a radius cutoff of 8 Å. Subsequently, the fraction of native contacts was computed throughout the trajectory for each condition. The average fraction of native contacts was derived from the last 500 frames of six independent replicates.

A salt bridge was considered to be formed if the distance between any of the oxygen atoms of acidic residues (ASP/GLU) and the nitrogen atoms of basic residues (ARG/LYS) was within a 3.2 Å cutoff distance in at least one frame. The percent occurrence of each salt bridge was computed based on the last 500 frames of the simulation, averaged across six independent replicates for each condition. Net charges were calculated from structure using an online p*K*_a_ prediction tool (https://www.ddl.unimi.it/vegaol/propka.htm) combined with the Henderson–Hasselbach equation.^65^ The “number of hydrogen bonds” present within the Fab structure was calculated in Gromacs and averaged over the last 50 ns of six independent replicates. Further feature engineering to combine features was not considered in the absence of any theoretical basis in order to avoid the risks associated with introducing artificial complexity and also overfitting the data.

### Pearson Correlation Analysis

Pearson correlation analysis was used first to investigate the linear/monotonic correlation between each single molecular feature calculated from MD, and the experimentally measured aggregation kinetics ln(*v*), and melting temperatures (*T*_m_). Pearson pairwise correlation between each of the single molecular features (*X* variables) and aggregation kinetics ln(*v*) or melting temperatures (*Y* variables) were calculated with the Python statistical package Pingouin.^66^ Spearman pairwise correlation was also performed to assist the interpretation of the monotonic relationship between the features.

### Feature Selection

Pearson correlation analysis and the Shapley additive explanations (SHAP) method^67^ (shap version 0.43.0, python version 3.11.6) were each examined for the feature selection process. SHAP uses classic Shapley values^68^ to rank the importance of the input features and signpost the redundancy between different features. The 17 molecular features shown in Table 2 were used as input variables and the SHAP value for each feature was calculated with the SHAP TreeExplainer.^69^

### Model Building

After the feature selection, a set of different statistical tests and predictive models were generated in this study, ranging from simple methods such as linear regression to more complex methods like partial least-squares (PLS), multiple linear regression (MLR), support vector regression (SVR), decision tree regression, and random forest regression. A *k*-fold cross validation was used to evaluate the performance of different models and select the best model among them. Generally, for small data sets the cross validation approach using the entire data set is more robust than retaining a single hold-out data set for validation purposes.^70^ The *k*-fold validation divided the data into *k* subsets, and in each trial of *k*-fold validation, one of the *k* subsets was used as the testing data, while the remaining *k* – 1 subsets were used as training data. This process was repeated *k* times, with each subset being used as the testing data once. A sensitivity analysis was also performed to configure the *k* value for the *k*-fold cross validation. After the *k*-fold cross validation, the best model was selected for further hyperparameter tuning and prediction purposes. All the model building was performed with scikit-learn package.^71^

**Scheme 1:**
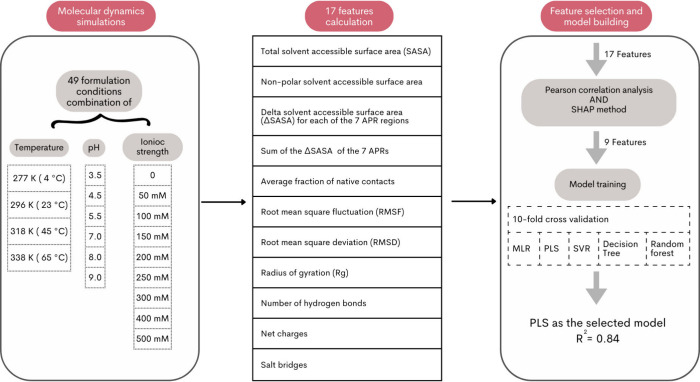
Overall Workflow for Predictive Model Building Employed in This Work

An overall workflow of the methodology, including molecular dynamics simulations, features calculation and selection, and predictive model building is shown in Scheme 1.

## Results and Discussion

### Overview of the Data Set Containing 49 Combinations of Temperature, pH, Ionic Strength, and Experimental Aggregation Kinetics

**Figure 1:**
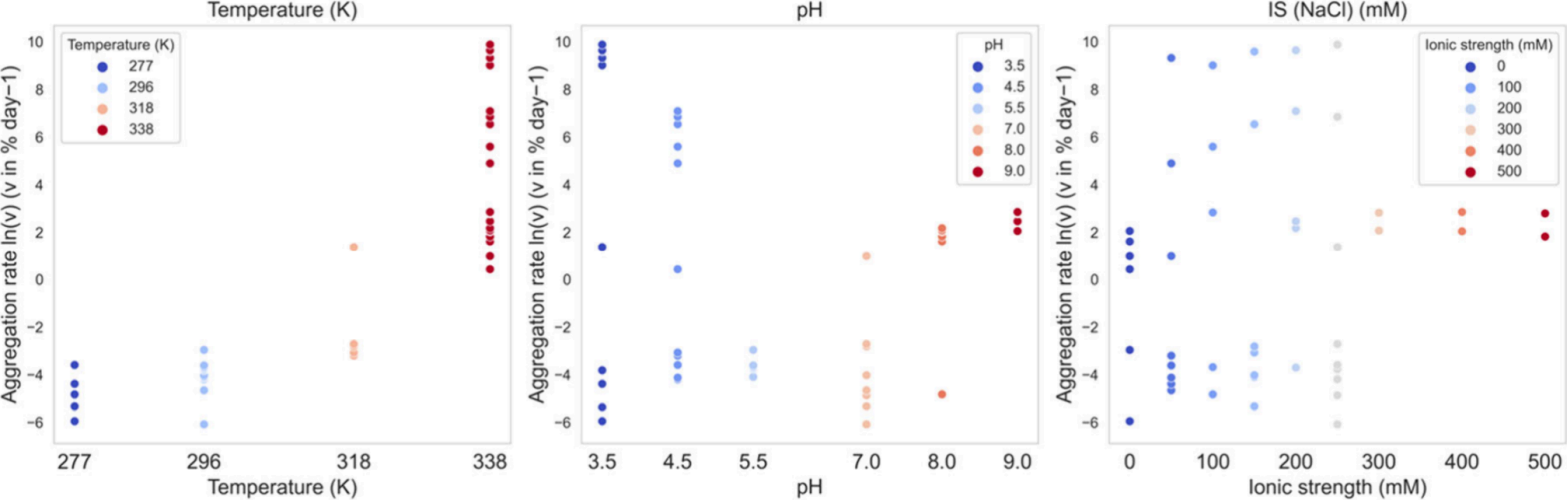
Scatter plots of the relationship between experimental aggregation kinetics and the 49 formulation conditions with varying temperature, pH, and ionic strength.

A statistical overview was first performed on the 49-condition data set in Table 1. The experimental aggregation kinetics were plotted against the temperature, pH, and ionic strength (Figure 1). This shows that the temperature had a strong impact on the aggregation kinetics, with higher temperatures leading to increased aggregation rates. Nevertheless, the relative impacts of pH and ionic strength can also be observed such that the aggregation rates varied in a wide range (ln(*v*) from 0 to 10) at a fixed temperature 338 K (65 °C). Additionally, when comparing the aggregation rates at temperatures 277 K (4 °C) and 296 K (23 °C), they did not display a significant difference, indicating that factors other than temperature, such as pH or ionic strength, also played a role. To address this argument further, a principal component analysis (Supporting Information) was performed on the entire data set to investigate the contribution of each variable. The loading plot from the PCA (Supporting Information, Figure S4) showed that pH and ionic strength contributed the most to the first principal component which explained 50% of the data, indicating that pH and ionic strength are important factors to consider during the model building process to predict the aggregation kinetics.

### RMSD and RMSF Analysis of MD Simulations

**Figure 2:**
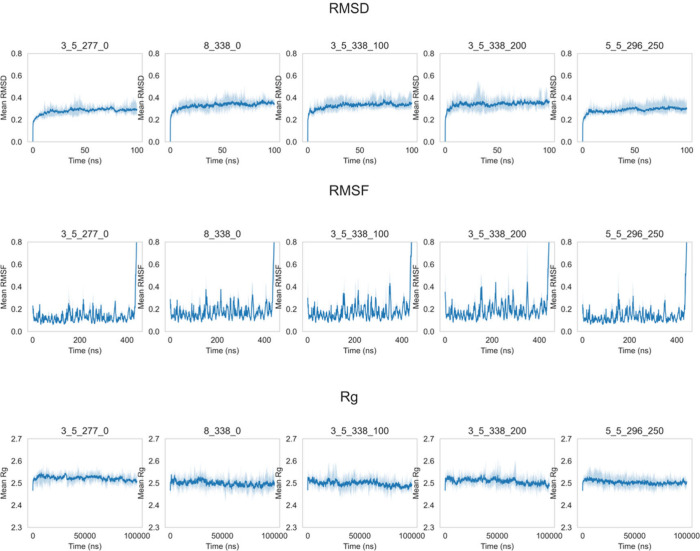
An example of the RMSD, RMSF, and radius of gyration (*R*_g_) for 5 out of 49 conditions. The shadowed background denotes the maximum and the minimum values, while the solid line is the average value

MD simulations were performed over 100 ns at conditions varying in temperature, pH and ionic strength to match 49 of the 105 conditions previously reported with experimental aggregation kinetics.^11^ The changes in RMSD, RMSF and radius of gyration (*R*_g_) over time are shown in Figure 2 (five conditions as examples) and in Figures S5–S7 (all of the conditions). These were further analyzed as normalized distribution functions as shown in Figures S8–S10 (Supporting Information). The distributions for RMSD and *R*_g_ demonstrated that the simulations were exploring multiple and distinct populations of structures and that the effect of temperature dominated in terms of the distribution of structures and their influence on RMSD and *R*_g_. Simulations with high temperatures displayed high RMSDs but low *R*_g_ values, indicating that the conformational changes were greatest at high temperatures. The flat appearance of the Fab A33 chains at the starting position tended to bend over during the simulations at a high temperature, which increased its compactness and resulted in low *R*_g_ values.

**Figure 3:**
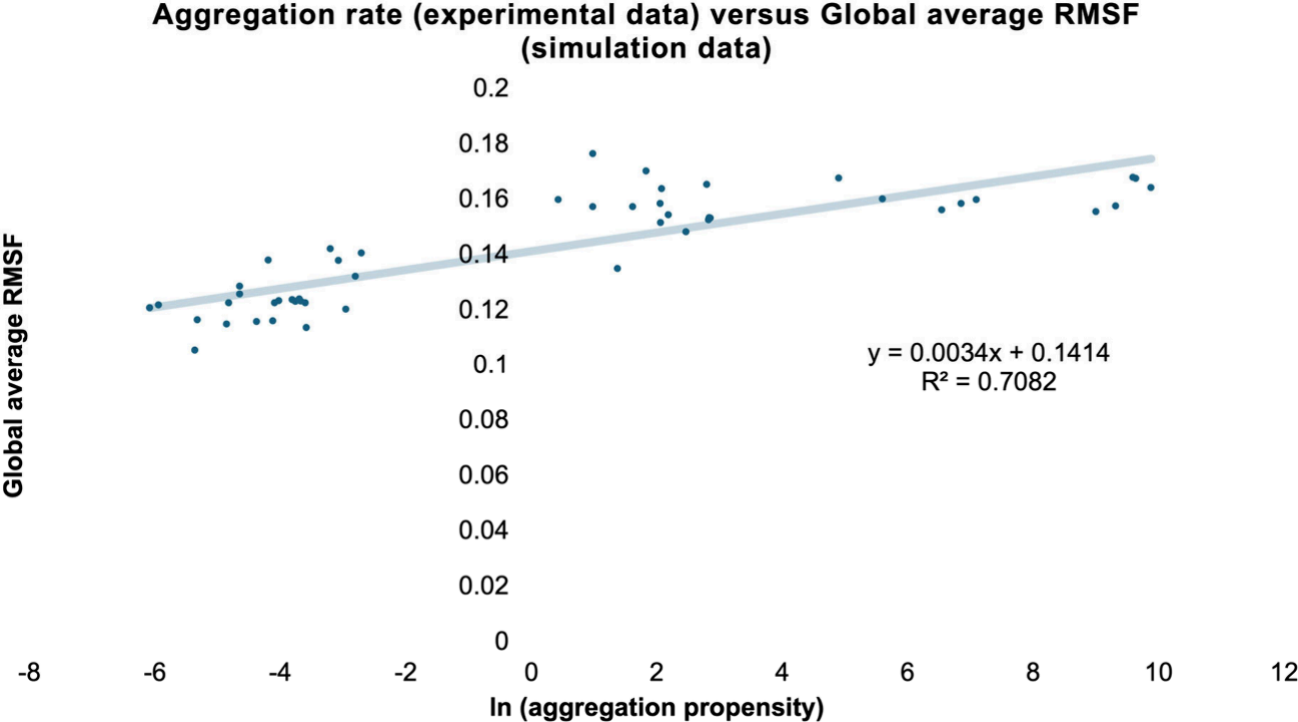
A simple linear regression model with an *R*^2^ of 0.71, built with the global average RMSF of 49 formulation conditions of Fab A33 and experimental aggregation kinetics. All of the Fab A33 simulations were conducted in aqueous solution consisting of varying pH and sodium chloride concentrations.

**Figure 4:**
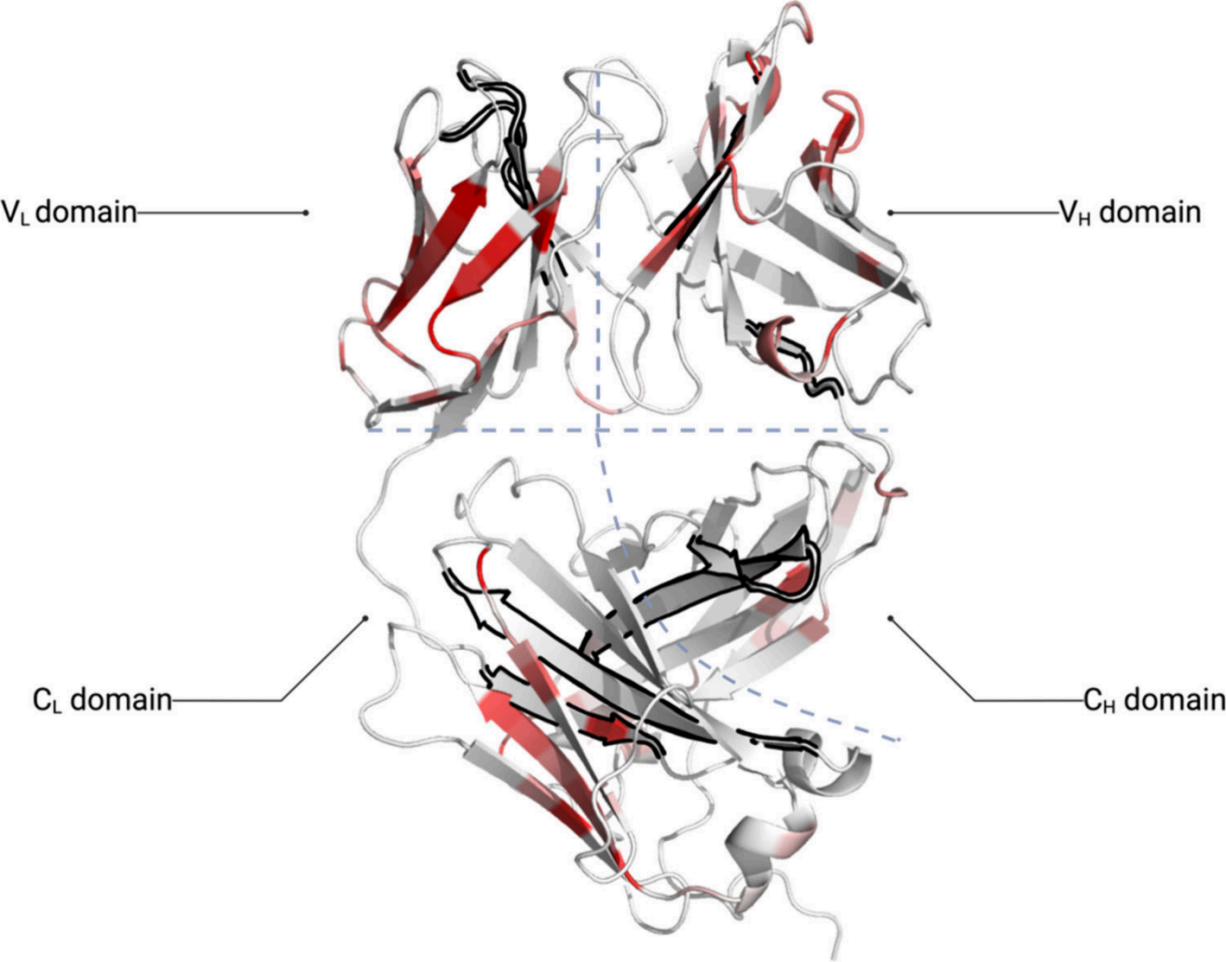
Crystal structure of Fab A33 color coded by the residue-level Pearson *R*^2^ values for the correlation between global RMSF and aggregation kinetics. Color varies from red (*R*^2^ = 0.7) to white (*R*^2^ = 0.65) to highlight only the highest *R*^2^ values. The seven predicted APRs are outlined in black. The image was generated in PyMOL.

A simple linear regression model was built between the global average RMSF of the 442 amino acid residues in the Fab molecule and the experimental aggregation kinetics data. The *R*^2^ values of the linear model reached 0.71 (Figure 3). Given this high *R*^2^, we also mapped the individual *R*^2^ for all residues (by performing a simple linear regression on the RMSF of each residue and experimental aggregation kinetics over the 49 formulation conditions) to the structure of Fab as shown in Figure 4. The protein structure is colored by *R*^2^ from red (*R*^2^ = 0.7) to white (*R*^2^ = 0.65) to focus on the regions that correlated better than average. Interestingly, several regions depicted in red appeared in surface exposed beta strands that protect one or more of the seven APR regions of the Fab A33 (black outline) from solvent. The APRs themselves are typically buried from solvent and so rarely depicted in red. This highlights the potential link between increased local fluctuations that could expose APR regions and lead to an increase in the aggregation kinetics. While this offers a potential mechanism for controlling aggregation kinetics, it does not include a range of other potentially contributing factors such as hydrophobicity, solvent accessibility, and surface charge distribution whose relative roles in the aggregation mechanism can be complex and also varying with formulation conditions. Therefore, it was worthwhile to explore a selection of different molecular features that describe the protein properties, with the potential to gain molecular insights into protein aggregation and increase the accuracy of the predictive tasks.

### Pearson Correlation Matrix between Molecular Features, Formulation Conditions, and Aggregation Kinetics

**Figure 5:**
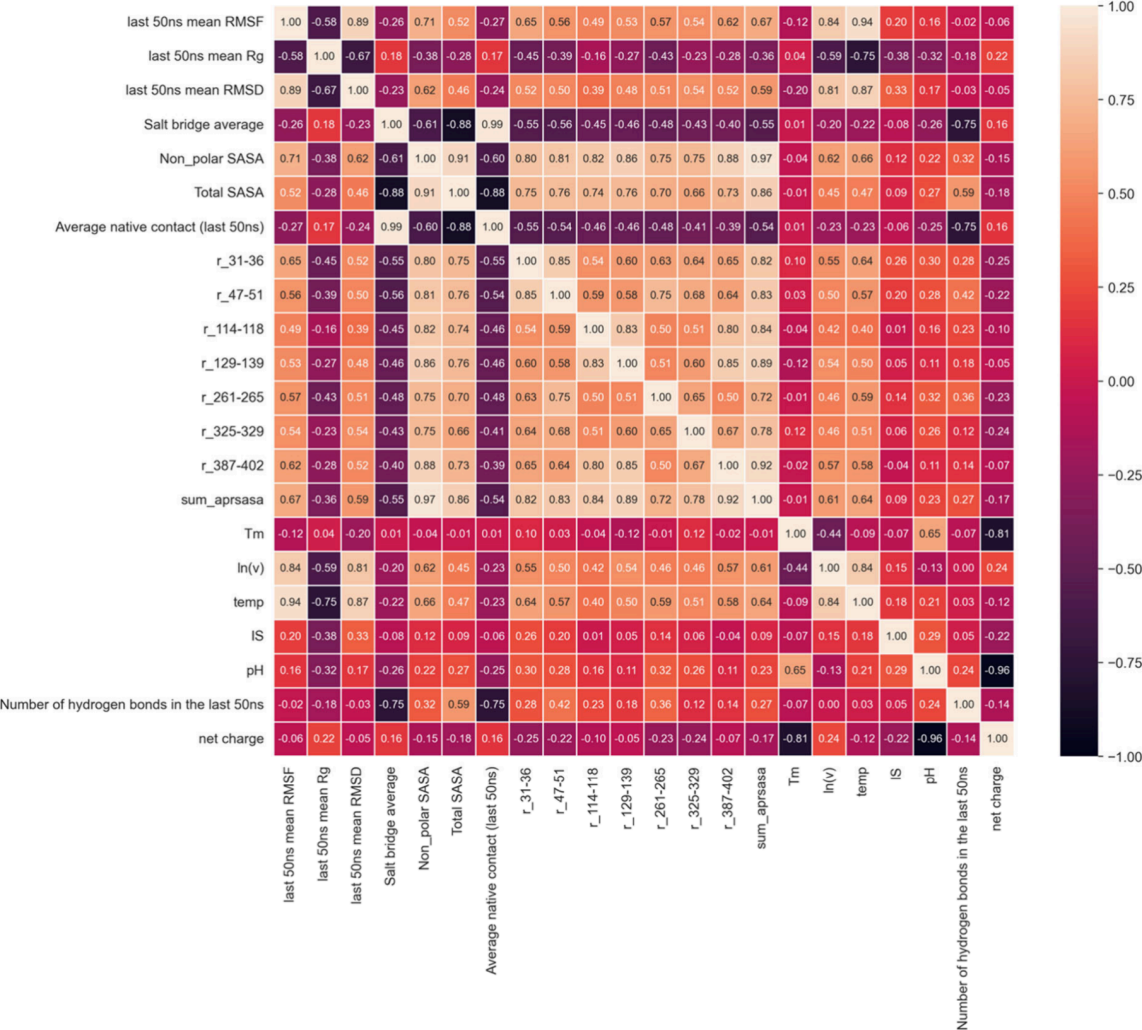
A heatmap of Pearson correlations (as *r* values) between all the variable features, experimental variables (as labels during the model building), and experimental data of melting temperature (*T*_m_) and aggregation kinetics (ln(*v*)). The color code shown on the right side with black indicates a strong negative correlation, whereas white or mild orange indicates a strong positive correlation. A detailed description for the names of the variables can be found in Table 2.

The correlation heatmap matrix between several experimental and calculated features, shown in Figure 5, ranges from strong negative (black) to strong positive (off-white) correlations. Eight features showed absolute values of the Pearson correlation coefficients with the experimental aggregation kinetics (ln(*v*)) of greater than 0.5 (i.e., last 50 ns mean RMSF, last 50 ns mean RMSD, last 50 ns mean *R*_g_, nonpolar SASA, r_31–36, r_129–139, r_387–402, sum_aprsasa). Global average RMSF had the highest Pearson correlation coefficient (*r*) with ln(*v*), reaching 0.84. The second highest Pearson correlation was for the last 50 ns of the mean RMSD with an r of 0.81. However, it is worth noting that these two features, as well as the experimental aggregation kinetics ln(*v*), all had a high correlation with temperature (RMSF, *r* = 0.94; RMSD, *r* = 0.87; ln(*v*), *r* = 0.84), making temperature a possible confounding factor in the correlation analysis. In this case, the experimental aggregation kinetics and the RMSF/RMSD might not be linked by only causal relationships.

Essentially, an increase in temperature is expected to increase RMSF and RMSD (Figures S8 and S10), across all residues within the protein, although by varying degrees dependent on the local structure. Mechanistically, some of these increases in structural dynamics could lead to an increased propensity for aggregation, but not necessarily all of them. Likewise, aggregation kinetics are expected to increase with temperature because of (i) the increased fluctuations of the native ensemble, leading to more aggregation-prone conformations, and (ii) the increased reaction kinetics resulting from faster diffusion in solution. It is therefore challenging to determine the direct contribution of protein dynamics (RMSF, RMSD) on aggregation kinetics. However, based on experimental evidence for Fab A33 and other proteins,^11,72^ we also expect that increased dynamics in particular regions of the protein lead to APR exposure and increased aggregation kinetics. Furthermore, for Fab A33, biophysical measurements of unfolding, and mechanistic modeling of aggregation kinetics have revealed that native ensemble dynamics have a critical influence on aggregation kinetics, and that Fab A33 aggregation was rate-limited by a partial-unfolding step under predominantly native conditions.^73^ For this reason, the influence of the temperature on diffusion rates is less critical to aggregation kinetics than the temperature-dependent structural dynamics within the native ensemble. Therefore, the correlation between RMSF and ln(*v*) is highly likely to be causal for at least a certain population of residues within the protein.

Between all of the feature variables, the average native contact had a strong negative Pearson correlation with total SASA, reaching *r* = −0.88. This can be explained by increases in SASA that occur when a protein starts to unfold or partially unfold or when the structure of the protein deviates from its native form. In turn, this also leads to a lower native contact fraction across the protein. All of the variables that are related to SASA were also well correlated as expected. The relationship between native contacts and the number of hydrogen bonds in the Pearson and Spearman correlation (Figure 5, Figure S11) showed a significant difference, with a strong negative Pearson correlation (*r* = −0.75), compared to a relatively weak Spearman’s correlation (ρ = −0.13) (Figure S11, Supporting Information). The unmatched outcome indicates that the correlation between native contact and the number of hydrogen bonds is approximately linear but not monotonic. A similar unmatched correlation between Pearson and Spearman analysis also happened between the average salt bridge occurrence and the number of hydrogen bonds (*r* = −0.75, ρ = −0.26). There were also cases where the two variables showed a strong Spearman correlation (Figure S11) but a relatively weak Pearson correlation, such as between the average salt bridge occurrence and mean RMSD (*r* = −0.23, ρ = −0.63), and global average RMSF and average native contact (*r* = −0.27, ρ = −0.83), indicating their correlation is nonlinear but monotonic. As for the others, the two correlation tests aligned well with each other.

Melting temperatures (*T*_m_) only showed a relatively high correlation with pH (*r* = 0.65) and net charge (*r* = −0.81). The dependence of *T*_m_ on pH has already been well characterized.^11^ The net charge was calculated using residue p*K*_a_ predictions from structure and the pH as mentioned earlier, making it a strong pH probe (correlation between pH and net charge: *r* = −0.96) and therefore also correlating strongly with *T*_m_. No other features appeared to correlate with the melting temperature. This first highlights our previous finding that *T*_m_ and aggregation kinetics do not correlate well across the four different temperatures for Fab A33.^11^ It also aligns with a previous finding where *T*_m_ did not show any observed correlation with 100 ns molecular dynamics simulations at 300–400 K (27–127 °C) (RMSD, RMSF, *R*_g_) for five mainly α-helical and five mainly β-structure small proteins.^41^ However, the results conflicted with another finding using 400 K MD simulations, which reported a high correlation between native contacts and *T*_m_.^42^ One possible explanation is that the latter simulations were able to initiate global unfolding processes of the proteins at 400 K (127 °C) and so were able to reflect changes relating to the *T*_m_. For our Fab A33 simulations the protein was not yet unfolded. This perhaps resulted in a lack of correlation between the fraction of native contacts and the melting temperature. Such findings also draw attention to the fact that aggregation kinetics for many proteins, including Fab A33, are highly dependent on native ensemble dynamics with transient local unfolding events, whereas melting temperatures report on global unfolding.

### Single Feature Predictor

Based on the correlation matrices discussed above and the domain knowledge for aggregation kinetics, it would be interesting to explore the predictive ability of each single feature for ranking the aggregation kinetics. The experimental aggregation kinetics of the 49 conditions were classified into three groups: high aggregation (ln(*v*) above 4), medium aggregation (ln(*v*) between 0 and 4), and low aggregation (ln(*v*) below 0). The aim here was not to build a regression function between the feature values and the aggregation kinetics but to evaluate the accuracy of using single features to classify conditions with a high risk level of aggregation (ln(*v*) above 4). Each of the 17 features was plotted against the experimental aggregation kinetics. Seventeen scatter plots with the molecular feature values as the *X* axis and experimental aggregation kinetics as the *Y* axis were generated with the experimental temperatures color-coded (Figure S12).

**Figure 6:**
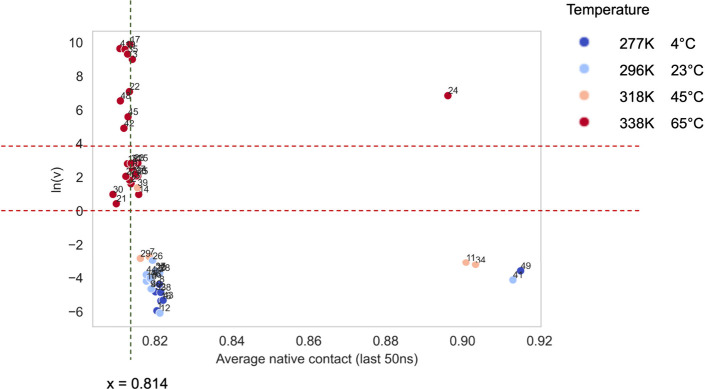
Correlations of Fab A33 aggregation kinetics with the average native contact feature. Experimental aggregation kinetics data were obtained previously.^11^ Data points are color coded by temperature. The two red dotted reference lines classify the aggregation data set into three groups: high aggregation (ln(*v*) above 4), medium aggregation (ln(*v*) between 0 and 4), and low aggregation (ln(*v*) below 0). The average native contacts for all the α-carbons in the protein in the last 50 ns for each simulation correlated with the aggregation kinetics data. The vertical gray line divides the red data points (ln(*v*) above 0) into two groups: left-hand side with high aggregation (ln(*v*) > 4) and average native contacts below 0.814; right-hand side with medium aggregation (0 < ln(*v*) < 4) and average native contacts above 0.814.

Among the 17 features, the average native contact feature could pick up high aggregation cases (ln(*v*) > 4) better than any other features (Figure 6). A total of 10 conditions had aggregation kinetics above 4, and the average native contact feature could pick up eight out of them, together with seven false positives that have aggregation rates between 0 and 4 (medium aggregation). This feature might be instructive to broadly classify aggregation rankings for industrial practical purposes. The results also aligned with previous efforts that aimed to use molecular features to classify aggregation risks^39^.^38^ Therefore, this approach could enable the screening of a large range of formulation conditions through simulations within a limited amount of time and signpost one or more of the most suitable formulation conditions. In practical terms, the top left corner of Figure 6 would be of specific interest to the industry to identify conditions with high aggregation risks. For example, in the current data set, any condition with a native contact value smaller than 0.814 had a possibility of 53% to have a high aggregation rate and almost certainly had at least a medium-level aggregation risk. Thus, the protein aggregation kinetics in different formulation conditions could be classified based on the calculation of the average native contact feature from MD simulations.

### Feature Selection

**Figure 7:**
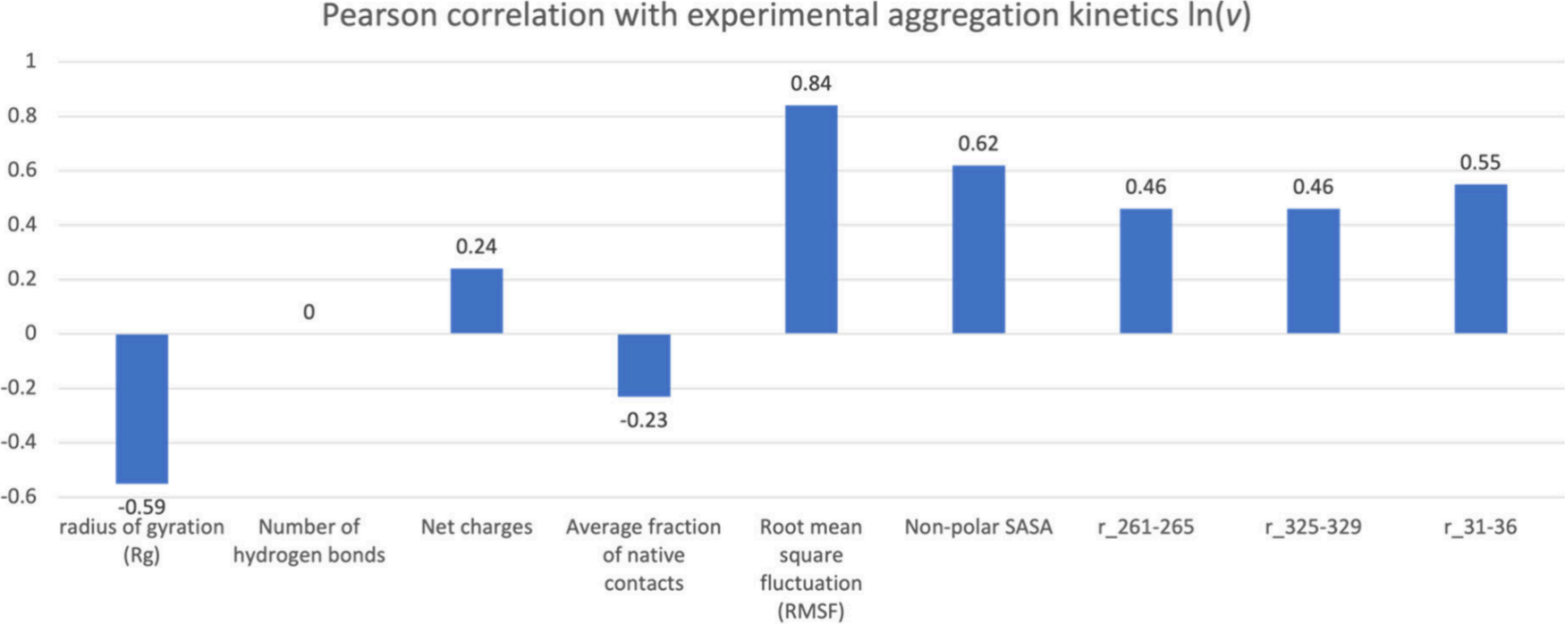
Nine features remained after the correlation coefficient feature selection process. The bar values are the Pearson correlation coefficients between the features and the experimental aggregation kinetics ln(*v*).

The single feature predictor approach was useful for identifying cases with high-risk aggregation. However, for a better understanding of the impact of pH and ionic strength on the aggregation kinetics, and to gain molecular-level insights into protein aggregation behavior, it remained beneficial to consider the contribution of various features, to explore different combinations of these features, and to build different statistical models with the feature combinations. First, we used the correlation heatmaps (Figure 5) to guide the feature selection for model building. Any two features that had a correlation coefficient higher than 0.8 indicated a higher chance of redundancy, and so the feature with the lower correlation with the experimental aggregation kinetics ln(*v*) was removed. Nine out of the 17 features remained after this correlation coefficient feature selection process (Figure 7). Among the nine features, net charges, number of hydrogen bonds, and average fraction of native contacts did not show strong Pearson correlation (|*r*| < 0.25) with the experimental aggregation kinetics, but we could not rule out the possibility that there could be a more complex correlation between them and so these features were kept.

**Figure 8:**
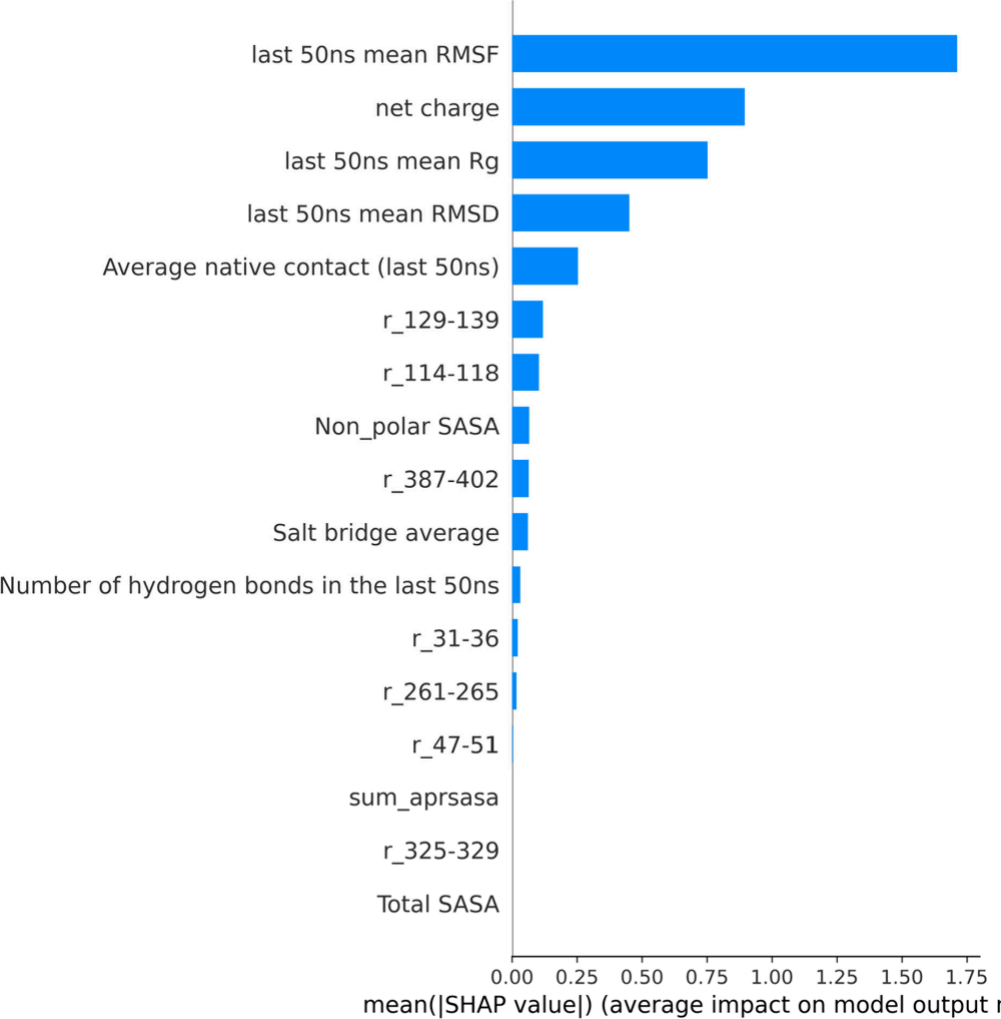
Relative contributions of 17 molecular features to the XGBboost model explained by SHAP as the average impact on the model output is shown as a bar chart. Longer bars indicate higher importance of the feature.

Another feature selection method used the SHAP TreeExplainer with the XGBRegressor model to rank high contribution features (Figure 8), and it was compared with the above correlation feature selection. Eight out of the top 13 features were the same from both selection methods. These indicated a mix of both surface properties (net charges, number of hydrogen bonds, SASA) and dynamics properties (global average RMSF, *R*_g_). The sum_aprsasa feature (i.e., sum of the ΔSASA of the 7 APRs), as indicated in both the correlation feature selection as well as in the SHAP method, did not show a strong correlation with the aggregation kinetics of the protein. This was interesting given that two ΔSASA of individual APRs were identified as important features in both feature selections, indicating that some APRs were more important for aggregation kinetics than others. This ranking of features from two feature selection methods also indirectly validated our choices during the single feature predictor section, where we purposefully selected several molecular features based on the domain knowledge to perform correlation analysis.

Mechanistically speaking, the eight overlapped features from both selection methods relate to protein hydrophobicity, unfolding status, surface charges, and solvent exposure of at least two of the predicted APRs. Previous studies have covered some of the features in terms of investigating protein aggregation in formulations,^38−41^ but none of them examined such a wide range of features as displayed in Table 2, nor did they specifically test the rankings of these features in terms of their contribution to aggregation kinetics.

In previous studies,^12,74^ small angle X-ray scattering (SAXS) and MD simulations in four different formulation conditions, were used to identify the regions (especially those close to APR regions) that appeared important for aggregation kinetics. The SAXS method, though, was able to display only an average structure of the Fab in each condition and therefore missed detailed information on the range of structural conformations accessed in the ensemble. The simulations conducted in four different conditions were able to pick up an APR on the V_H_ domain that had a strong impact on the aggregation kinetics.^12^ Furthermore, with the 49 formulation conditions explored in this study, we were able to identify more regions that might be relevant to aggregation kinetics. The Pearson and SHAP analysis confirmed the importance of some APRs (residues 129–139, C_L_, and residues 261–265, V_H_) identified previously but also highlighted that the APRs at residues 114–118 (C_L_), 387–402 (C_H_1) and residues 31–36 (V_L_) might also contribute to the aggregation kinetics. Figure 4 depicts the Fab regions in red as those whereby their RMSF values had a high correlation with the experimental aggregation rates (*R*^2^ > 0.65), and many were found close to the APR regions (black outline). Taken together, the APRs identified both through the RMSF correlations with aggregation kinetics (Figure 3), and also flagged as important by SHAP (Figure 8), are the most likely to be involved in the aggregation mechanism.

The good overlap of eight features identified by both approaches brings confidence to their importance. The SHAP method ranking is difficult to interpret mechanistically, and the outcome may also vary depending on the mathematical models the user selects to calculate the ranking; however, the correlation analysis was easier to interpret. Therefore, the nine features from the correlation selection were used for model building.

### Advanced Predictive Models for Estimating Aggregation Kinetics

After selecting the nine features based on the correlation analysis (Figure 7), they were used as inputs for more complex predictive model building to estimate the aggregation kinetics. The main steps of the model building process involved first tuning the hyperparameter for the *k*-fold cross validation, then using the *k*-fold cross validation to select the best performing model among a range of different mathematical models, and at last training the whole data set with the selected best performing model to offer prediction. The choice of the hyperparameter *k* for the *k*-fold cross validation used a sensitivity analysis testing the fold number between 2 and 31. The mean squared error (MSE) generated by each fold was compared to that from leave-one-out cross-validation (LOOCV), the reference baseline. Most *k* values had similar performance with LOOCV (MSE of 6.86), and *k* = 10 with an MSE of 6.81 was chosen since it is the most common configuration. 10-fold cross validation was thus selected to evaluate different mathematical models and compare their performance.

**Figure 9:**
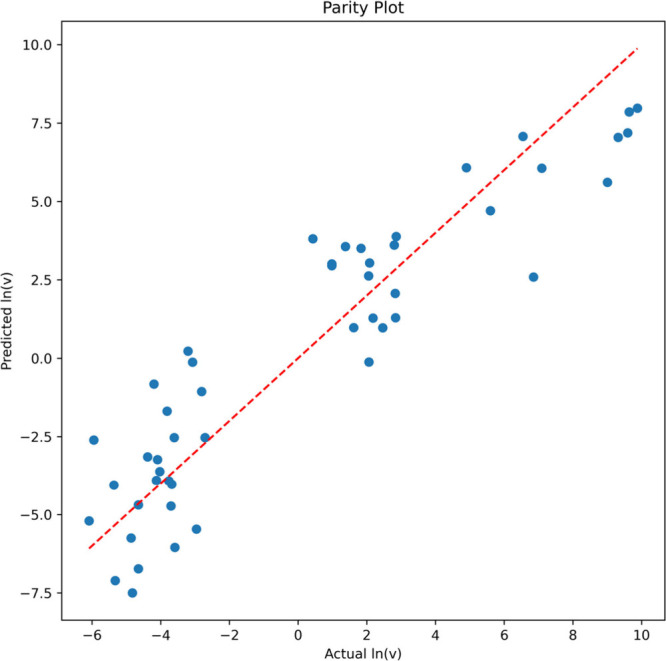
A parity plot between predictive values and actual values from the PLS regression model prediction with an *R*^2^ of 0.84.

A range of regression models were chosen with the aim of building models that could be interpreted to provide insights into protein aggregation. These included multiple linear regression (MLR), partial least-squares (PLS), support vector regression (SVR), decision tree regression, and random forest regression from scikit-learn package^71^ that were examined with 10-fold cross validation (10-fold CV) to estimate how well each model would perform. An ideal test (LOOCV) using the same regression models as a reference test was also performed. The mean MSE, maximum MSE, and the standard deviation of MSE were calculated for each model in both tests and could be found in Table S1. The random forest regression had the lowest MSE in both the ideal test (MSE = 3.89) and the 10-fold CV test (MSE = 3.74) but also had a high standard deviation in both tests (LOOCV with std = 12.23 and 10-fold CV test with std = 4.76), indicating that its performance was not stable. The same happened for decision tree regression. By contrast, PLS had the highest evaluation *R*^2^ of 0.58 (training data set divided with 10-fold CV) in the remaining models, the lowest mean MSE in LOOCV (MSE = 5.64), and a low variability in the 10-fold cross validation test (std = 3.29). Therefore, the PLS regression model was selected as the predictive model that was then trained on the whole data set. The final *R*^2^ of the model reached 0.84, and a parity plot showing the predicted values versus the actual experimental aggregation kinetics is shown in Figure 9.

In addition to predicting the aggregation kinetics, the same workflow was applied to build predictive models for the experimentally determined fraction of unfolding ln(*f)* of Fab A33. The same 17 molecular features were used, and a series of statistical analyses and model building were performed, including Pearson and Spearman correlation, PLS, MLR, decision tree regression, random forest regression, and *k*-fold cross-validation analysis. The fraction of unfolding ln(*f*), like aggregation kinetics ln(*v*), was temperature-dependent and had a high correlation with temperature-dependent molecular features such as mean RMSD, global average RMSF, and mean *R*_g_. The best model performance was from a multiple linear regression, with *R*^2^ = 0.88. This was not unexpected given the close relationship between ln(*v*) and ln(*f*). All the figures and statistical analysis can be found in the Supporting Information (Figures S13–S16).

As a summary, the workflow included the application of the *k*-fold cross validation to first find a suitable candidate for model building, and PLS was selected to build models for this data set for aggregation prediction. Based on the current model building process, further work can be done to fine-tune the models in the future, as more formulations get added to the data set, while also continuing to explore the full range of models when repeating the model selection and performance analysis.

## Conclusion

The MD simulation study of Fab A33 was carried out over 49 solution conditions and compared to experimental aggregation kinetics and thermostability. The RMSD, RMSF, and *R*_g_ in MD simulations and the aggregation kinetics were all highly dependent on temperature, making it challenging to separate out molecular fluctuations that accelerate aggregation mechanistically, from noncausal co-dependence on temperature. As the conditions were also varied using pH and ionic strength, which had a smaller but significant impact on aggregation kinetics, the relationship between a range of molecular fluctuations and other structural features and aggregation kinetics could be further evaluated by building more complex predictive models. The surface properties, electrostatic interactions, dynamics properties, and topological information were calculated for a total of 17 features generated from molecular dynamics simulations under various formulation conditions.

SHAP analysis together with Pearson correlation were used to guide feature selection and combination and to elucidate the features that contributed most to predictive models for aggregation kinetics. This analysis, along with matching the residue-level RMSF with the experimental aggregation kinetics, also offers insight into specific residue-level regions of the protein whose molecular dynamics may lead to increased levels of aggregation across a range of antibody drug formulations.

Based on this feature engineering, different types of predictive regression models were constructed, from simple models such as the linear regression model to sophisticated ones like PLS, MLR, decision tree, and random forest. The best model for aggregation kinetics prediction made use of the PLS regression model with an *R*^2^ of 0.84. The best model for predicting the fraction of protein unfolded was a multiple linear regression model with *R*^2^ = 0.88. These models demonstrate their potential to guide the early detection of aggregation-prone formulation conditions, provide unique information gained from dynamics insights for antibody screening and design, and offer potential solutions for increasing developmental characteristics for pharmaceutical proteins. Based on the workflow established with this Fab fragment, it will be interesting to extend the approach to include the effects of excipients, to evaluate full antibodies, and also to determine whether the approach can be further streamlined by using coarse-grained simulations.

## Supporting Information Available

Fab A33 sequence information; benchmark of different force fields of all-atom simulations; principal component analysis on the 49 condition data set; plots of RMSD, RMSF, and radius of gyration over 100 ns for 49 conditions with 6 replicas each; distribution analysis of RMSD and radius of gyration (*R*_g_) at four different temperatures; residue-level RMSF over a range of selected conditions; heatmap of Spearman correlations between all the variable features and experimental variables; 17 scatter plots of the molecular feature values versus the experimental aggregation kinetics; model building for predicting aggregation kinetics; model building for predicting fraction of unfolding (PDF)

## References

[ref1] Raybould M. I. J.; Marks C.; Krawczyk K.; Taddese B.; Nowak J.; Lewis A. P.; Bujotzek A.; Shi J.; Deane C. M. Five computational developability guidelines for therapeutic antibody profiling. P Natl. Acad. Sci. Usa 2019, 116, 4025–4030. 10.1073/pnas.1810576116. PMC6410772 30765520

[ref2] Dyson M. R.; Masters E.; Pazeraitis D.; Perera R. L.; Syrjanen J. L.; Surade S.; Thorsteinson N.; Parthiban K.; Jones P. C.; Sattar M.; Wozniak-Knopp G.; Rueker F.; Leah R.; McCafferty J. Beyond affinity: selection of antibody variants with optimal biophysical properties and reduced immunogenicity from mammalian display libraries. MAbs 2020, 12, 1829335 10.1080/19420862.2020.1829335. 33103593 PMC7592150

[ref3] Jain T.; Sun T.; Durand S.; Hall A.; Houston N. R.; Nett J. H.; Sharkey B.; Bobrowicz B.; Caffry I.; Yu Y.; Cao Y.; Lynaugh H.; Brown M.; Baruah H.; Gray L. T.; Krauland E. M.; Xu Y.; Vásquez M.; Wittrup K. D. Biophysical properties of the clinical-stage antibody landscape. Proc. National Acad. Sci. 2017, 114, 944–949. 10.1073/pnas.1616408114. PMC5293111 28096333

[ref4] Bailly M.; Mieczkowski C.; Juan V.; Metwally E.; Tomazela D.; Baker J.; Uchida M.; Kofman E.; Raoufi F.; Motlagh S.; Yu Y.; Park J.; Raghava S.; Welsh J.; Rauscher M.; Raghunathan G.; Hsieh M.; Chen Y.-L.; Nguyen H. T.; Nguyen N.; Cipriano D.; Fayadat-Dilman L. Predicting Antibody Developability Profiles Through Early Stage Discovery Screening. MAbs 2020, 12, 1743053 10.1080/19420862.2020.1743053. 32249670 PMC7153844

[ref5] Svilenov H. L.; Arosio P.; Menzen T.; Tessier P.; Sormanni P. Approaches to expand the conventional toolbox for discovery and selection of antibodies with drug-like physicochemical properties. MAbs 2023, 15, 2164459 10.1080/19420862.2022.2164459. 36629855 PMC9839375

[ref6] Wang W. Instability, stabilization, and formulation of liquid protein pharmaceuticals. Int. J. Pharmaceut 1999, 185, 129–188. 10.1016/S0378-5173(99)00152-0. 10460913

[ref7] Wang W.; Ohtake S. Science and Art of Protein Formulation Development. Int. J. Pharmaceut 2019, 568, 118505 10.1016/j.ijpharm.2019.118505. 31306712

[ref8] Jarasch A.; Koll H.; Regula J. T.; Bader M.; Papadimitriou A.; Kettenberger H. Developability Assessment During the Selection of Novel Therapeutic Antibodies. J. Pharm. Sci. 2015, 104, 1885–1898. 10.1002/jps.24430. 25821140

[ref9] Andrews J. M.; Roberts C. J. A Lumry–Eyring Nucleated Polymerization Model of Protein Aggregation Kinetics: 1. Aggregation with Pre-Equilibrated Unfolding. J. Phys. Chem. B 2007, 111, 7897–7913. 10.1021/jp070212j. 17571872

[ref10] Wang W.; Roberts C. J. Protein aggregation – Mechanisms, detection, and control. Int. J. Pharmaceut 2018, 550, 251–268. 10.1016/j.ijpharm.2018.08.043. 30145245

[ref11] Chakroun N.; Hilton D.; Ahmad S. S.; Platt G. W.; Dalby P. A. Mapping the Aggregation Kinetics of a Therapeutic Antibody Fragment. Mol. Pharmaceut 2016, 13, 307–319. 10.1021/acs.molpharmaceut.5b00387. 26692229

[ref12] Zhang C.; Codina N.; Tang J.; Yu H.; Chakroun N.; Kozielski F.; Dalby P. A. Comparison of the pH- and thermally-induced fluctuations of a therapeutic antibody Fab fragment by molecular dynamics simulation. Comput. Struct Biotechnology J. 2021, 19, 2726–2741. 10.1016/j.csbj.2021.05.005. PMC8131956 34093988

[ref13] Song J. G.; Lee S. H.; Han H.-K. The stabilization of biopharmaceuticals: current understanding and future perspectives. J. Pharm. Investig. 2017, 47, 475–496. 10.1007/s40005-017-0341-9.

[ref14] Roberts C. J. Therapeutic protein aggregation: mechanisms, design, and control. Trends Biotechnol 2014, 32, 372–380. 10.1016/j.tibtech.2014.05.005. 24908382 PMC4146573

[ref15] Berner C.; Menzen T.; Winter G.; Svilenov H. L. Combining Unfolding Reversibility Studies and Molecular Dynamics Simulations to Select Aggregation-Resistant Antibodies. Mol. Pharmaceut 2021, 18, 2242–2253. 10.1021/acs.molpharmaceut.1c00017. 33928776

[ref16] Manning M. C.; Chou D. K.; Murphy B. M.; Payne R. W.; Katayama D. S. Stability of Protein Pharmaceuticals: An Update. Pharm. Res. 2010, 27, 544–575. 10.1007/s11095-009-0045-6. 20143256

[ref17] Brader M. L.; Estey T.; Bai S.; Alston R. W.; Lucas K. K.; Lantz S.; Landsman P.; Maloney K. M. Examination of Thermal Unfolding and Aggregation Profiles of a Series of Developable Therapeutic Monoclonal Antibodies. Mol. Pharmaceutics 2015, 12, 1005–1017. 10.1021/mp400666b. 25687223

[ref18] Clarkson B. R.; Schön A.; Freire E. Conformational stability and self-association equilibrium in biologics. Drug Discov, Today 2016, 21, 342–347. 10.1016/j.drudis.2015.11.007. 26608889 PMC4761488

[ref19] Vázquez-Rey M.; Lang D. A. Aggregates in monoclonal antibody manufacturing processes. Biotechnol. Bioeng. 2011, 108, 1494–1508. 10.1002/bit.23155. 21480193

[ref20] Yadav S.; Laue T. M.; Kalonia D. S.; Singh S. N.; Shire S. J. The Influence of Charge Distribution on Self-Association and Viscosity Behavior of Monoclonal Antibody Solutions. Mol. Pharmaceutics 2012, 9, 791–802. 10.1021/mp200566k. 22352470

[ref21] Geng S. B.; Cheung J. K.; Narasimhan C.; Shameem M.; Tessier P. M. Improving Monoclonal Antibody Selection and Engineering using Measurements of Colloidal Protein Interactions. J. Pharm. Sci. 2014, 103, 3356–3363. 10.1002/jps.24130. 25209466 PMC4206634

[ref22] Powers E. T.; Balch W. E. Diversity in the origins of proteostasis networks — a driver for protein function in evolution. Nat. Rev. Mol. Cell Biol. 2013, 14, 237–248. 10.1038/nrm3542. 23463216 PMC3718298

[ref23] Gamage C. L. D.; Weis D. D.; Walters B. T. Identification of Agitation-Induced Unfolding Events Causing Aggregation of Monoclonal Antibodies Using Hydrogen Exchange-Mass Spectrometry. J. Pharm. Sci. 2022, 111, 2210–2216. 10.1016/j.xphs.2022.05.002. 35533783

[ref24] Li W.; Prabakaran P.; Chen W.; Zhu Z.; Feng Y.; Dimitrov D. S. Antibody Aggregation: Insights from Sequence and Structure. Antibodies 2016, 5, 19 10.3390/antib5030019. 31558000 PMC6698864

[ref25] Zhang C.; Bye J. W.; Lui L. H.; Zhang H.; Hales J.; Brocchini S.; Curtis R. A.; Dalby P. A. Enhanced Thermal Stability and Reduced Aggregation in an Antibody Fab Fragment at Elevated Concentrations. Mol. Pharmaceutics 2023, 20, 2650–2661. 10.1021/acs.molpharmaceut.3c00081. PMC10155210 37040431

[ref26] Conchillo-Solé O.; de Groot N. S.; Avilés F. X.; Vendrell J.; Daura X.; Ventura S. AGGRESCAN: a server for the prediction and evaluation of “hot spots” of aggregation in polypeptides. BMC Bioinf. 2007, 8, 65 10.1186/1471-2105-8-65. PMC1828741 17324296

[ref27] Fernandez-Escamilla A.-M.; Rousseau F.; Schymkowitz J.; Serrano L. Prediction of sequence-dependent and mutational effects on the aggregation of peptides and proteins. Nat. Biotechnol. 2004, 22, 1302–1306. 10.1038/nbt1012. 15361882

[ref28] Walsh I.; Seno F.; Tosatto S. C. E.; Trovato A. PASTA 2.0: an improved server for protein aggregation prediction. Nucleic Acids Res. 2014, 42, W301–W307. 10.1093/nar/gku399. 24848016 PMC4086119

[ref29] Garbuzynskiy S. O.; Lobanov M.Yu.; Galzitskaya O. V. FoldAmyloid: a method of prediction of amyloidogenic regions from protein sequence. Bioinformatics 2010, 26, 326–332. 10.1093/bioinformatics/btp691. 20019059

[ref30] Tartaglia G. G.; Cavalli A.; Pellarin R.; Caflisch A. Prediction of aggregation rate and aggregation-prone segments in polypeptide sequences. Protein Sci. 2005, 14, 2723–2734. 10.1110/ps.051471205. 16195556 PMC2253302

[ref31] Sankar K.; Krystek S. R.; Carl S. M.; Day T.; Maier J. K. X. AggScore: Prediction of aggregation-prone regions in proteins based on the distribution of surface patches. Proteins Struct Funct Bioinform 2018, 86, 1147–1156. 10.1002/prot.25594. 30168197

[ref32] Zambrano R.; Jamroz M.; Szczasiuk A.; Pujols J.; Kmiecik S.; Ventura S. AGGRESCAN3D (A3D): server for prediction of aggregation properties of protein structures. Nucleic Acids Res. 2015, 43, W306–W313. 10.1093/nar/gkv359. 25883144 PMC4489226

[ref33] Voynov V.; Chennamsetty N.; Kayser V.; Helk B.; Trout B. L. Predictive tools for stabilization of therapeutic proteins. MAbs 2009, 1, 580–582. 10.4161/mabs.1.6.9773. 20068399 PMC2791315

[ref34] van der Kant R.; van Durme J.; Rousseau F.; Schymkowitz J. SolubiS: Optimizing Protein Solubility by Minimal Point Mutations. In Protein Misfolding Diseases, Methods and Protocols; Methods Molecular Biology, Vol. 1873; Humana Press, 2018; pp 317–333, 10.1007/978-1-4939-8820-4_21. 30341620

[ref35] Sormanni P.; Aprile F. A.; Vendruscolo M. The CamSol Method of Rational Design of Protein Mutants with Enhanced Solubility. J. Mol. Biol. 2015, 427, 478–490. 10.1016/j.jmb.2014.09.026. 25451785

[ref36] Lauer T. M.; Agrawal N. J.; Chennamsetty N.; Egodage K.; Helk B.; Trout B. L. Developability Index: A Rapid In Silico Tool for the Screening of Antibody Aggregation Propensity. J. Pharm. Sci. 2012, 101, 102–115. 10.1002/jps.22758. 21935950

[ref37] Obrezanova O.; Arnell A.; de la Cuesta R. G.; Berthelot M. E.; Gallagher T. R.; Zurdo J.; Stallwood Y. Aggregation risk prediction for antibodies and its application to biotherapeutic development. mAbs 2015, 7, 352–363. 10.1080/19420862.2015.1007828. 25760769 PMC4622581

[ref38] Lai P.-K.; Fernando A.; Cloutier T. K.; Gokarn Y.; Zhang J.; Schwenger W.; Chari R.; Calero-Rubio C.; Trout B. L. Machine Learning Applied to Determine the Molecular Descriptors Responsible for the Viscosity Behavior of Concentrated Therapeutic Antibodies. Mol. Pharmaceutics 2021, 18, 1167–1175. 10.1021/acs.molpharmaceut.0c01073. 33450157

[ref39] Lai P.-K.; Fernando A.; Cloutier T. K.; Kingsbury J. S.; Gokarn Y.; Halloran K. T.; Calero-Rubio C.; Trout B. L. Machine Learning Feature Selection for Predicting High Concentration Therapeutic Antibody Aggregation. J. Pharm. Sci. 2021, 110, 1583–1591. 10.1016/j.xphs.2020.12.014. 33346034

[ref40] Lai P.-K.; Gallegos A.; Mody N.; Sathish H. A.; Trout B. L. Machine learning prediction of antibody aggregation and viscosity for high concentration formulation development of protein therapeutics. MAbs 2022, 14, 2026208 10.1080/19420862.2022.2026208. 35075980 PMC8794240

[ref41] Jana K.; Mehra R.; Dehury B.; Blundell T. L.; Kepp K. P. Common mechanism of thermostability in small α- and β-proteins studied by molecular dynamics. Proteins: Struct. Funct., Bioinform. 2020, 88, 1233–1250. 10.1002/prot.25897. 32368818

[ref42] Bekker G.; Ma B.; Kamiya N. Thermal stability of single-domain antibodies estimated by molecular dynamics simulations. Protein Sci. 2019, 28, 429–438. 10.1002/pro.3546. 30394618 PMC6319760

[ref43] Cloutier T.; Sudrik C.; Mody N.; Sathish H. A.; Trout B. L. Molecular Computations of Preferential Interaction Coefficients of IgG1 Monoclonal Antibodies with Sorbitol, Sucrose, and Trehalose and the Impact of These Excipients on Aggregation and Viscosity. Mol. Pharmaceutics 2019, 16, 3657–3664. 10.1021/acs.molpharmaceut.9b00545. 31276620

[ref44] Fitzpatrick A. W.; Knowles T. P. J.; Waudby C. A.; Vendruscolo M.; Dobson C. M. Inversion of the Balance between Hydrophobic and Hydrogen Bonding Interactions in Protein Folding and Aggregation. PLoS Comput. Biol. 2011, 7, e1002169 10.1371/journal.pcbi.1002169. 22022239 PMC3192805

[ref45] Stock P.; Monroe J. I.; Utzig T.; Smith D. J.; Shell M. S.; Valtiner M. Unraveling Hydrophobic Interactions at the Molecular Scale Using Force Spectroscopy and Molecular Dynamics Simulations. ACS Nano 2017, 11, 2586–2597. 10.1021/acsnano.6b06360. 28267918

[ref46] Rospiccio M.; Casucci P.; Arsiccio A.; Udrescu C.; Pisano R. Mechanistic Investigation of tert-Butanol’s Impact on Biopharmaceutical Formulations: When Experiments Meet Molecular Dynamics. Mol. Pharmaceutics 2023, 20, 3975–3986. 10.1021/acs.molpharmaceut.3c00125. PMC10410665 37435823

[ref47] Jha P. K.; Larson R. G. Assessing the Efficiency of Polymeric Excipients by Atomistic Molecular Dynamics Simulations. Mol. Pharmaceutics 2014, 11, 1676–1686. 10.1021/mp500068w. 24708235

[ref48] Li C.-X.; Wang H.-B.; Oppong D.; Wang J.-X.; Chen J.-F.; Le Y. Excipient-Assisted Vinpocetine Nanoparticles: Experiments and Molecular Dynamic Simulations. Mol. Pharmaceutics 2014, 11, 4023–4035. 10.1021/mp500045t. 25244002

[ref49] Saurabh S.; Zhang Q.; Seddon J. M.; Lu J. R.; Kalonia C.; Bresme F. Unraveling the Microscopic Mechanism of Molecular Ion Interaction with Monoclonal Antibodies: Impact on Protein Aggregation. Mol. Pharm. 2024, 21, 1285 10.1021/acs.molpharmaceut.3c00963. 38345400 PMC10915798

[ref50] Tosstorff A.; Svilenov H.; Peters G. H. J.; Harris P.; Winter G. Structure-based discovery of a new protein-aggregation breaking excipient. Eur. J. Pharm. Biopharm. 2019, 144, 207–216. 10.1016/j.ejpb.2019.09.010. 31521717

[ref51] Rospiccio M.; Arsiccio A.; Winter G.; Pisano R. The Role of Cyclodextrins against Interface-Induced Denaturation in Pharmaceutical Formulations: A Molecular Dynamics Approach. Mol. Pharmaceutics 2021, 18, 2322–2333. 10.1021/acs.molpharmaceut.1c00135. PMC8289300 33999634

[ref52] Ko S. K.; Björkengren G.; Berner C.; Winter G.; Harris P.; Peters G. H. J. Combining Molecular Dynamics Simulations and Biophysical Characterization to Investigate Protein-Specific Excipient Effects on Reteplase during Freeze Drying. Pharmaceutics 2023, 15, 1854 10.3390/pharmaceutics15071854. 37514040 PMC10384596

[ref53] King T. E.; Humphrey J. R.; Laughton C. A.; Thomas N. R.; Hirst J. D. Optimizing Excipient Properties to Prevent Aggregation in Biopharmaceutical Formulations. J. Chem. Inf. Model. 2024, 64, 265–275. 10.1021/acs.jcim.3c01898. 38113509 PMC10777730

[ref54] Venanzi N. A. E.; Basciu A.; Vargiu A. V.; Kiparissides A.; Dalby P. A.; Dikicioglu D. Machine Learning Integrating Protein Structure, Sequence, and Dynamics to Predict the Enzyme Activity of Bovine Enterokinase Variants. J. Chem. Inf. Model. 2024, 64, 2681 10.1021/acs.jcim.3c00999. 38386417 PMC11005043

[ref55] Zhang H.; Yang Y.; Zhang C.; Farid S. S.; Dalby P. A. Machine learning reveals hidden stability code in protein native fluorescence. Comput. Struct Biotechnology J. 2021, 19, 2750–2760. 10.1016/j.csbj.2021.04.047. PMC8131987 34093990

[ref56] Emily M.; Talvas A.; Delamarche C. MetAmyl: A METa-Predictor for AMYLoid Proteins. PLoS One 2013, 8, e79722 10.1371/journal.pone.0079722. 24260292 PMC3834037

[ref57] Tang J.; Zhang C.; Dalby P.; Kozielski F. The structure of the humanised A33 Fab C226S variant, an immunotherapy candidate for colorectal cancer. BioRxiv 2022, 2022.06.21.497004 10.1101/2022.06.21.497004.

[ref58] Abraham M. J.; Murtola T.; Schulz R.; Páll S.; Smith J. C.; Hess B.; Lindahl E. GROMACS: High performance molecular simulations through multi-level parallelism from laptops to supercomputers. SoftwareX 2015, 1, 19–25. 10.1016/j.softx.2015.06.001.

[ref59] Jorgensen W. L.; Maxwell D. S.; Tirado-Rives J. Development and Testing of the OPLS All-Atom Force Field on Conformational Energetics and Properties of Organic Liquids. J. Am. Chem. Soc. 1996, 118, 11225–11236. 10.1021/ja9621760.

[ref60] Cornell W.D.; Cieplak P.; Bayly C.I.; Gould I.R.; Merz K.M.; Ferguson D.M.; Spellmeyer D.C.; Fox T.; Caldwell J.W.; Kollman P.A. A Second Generation Force Field for the Simulation of Proteins, Nucleic Acids, and Organic Molecules. J. Am Chem. Soc. 1995, 117, 5179 10.1021/ja00124a002.

[ref61] Hornak V.; Abel R.; Okur A.; Strockbine B.; Roitberg A.; Simmerling C. Comparison of multiple Amber force fields and development of improved protein backbone parameters. Proteins Struct Funct Bioinform 2006, 65, 712–725. 10.1002/prot.21123. PMC4805110 16981200

[ref62] Kollman P. A. Advances and Continuing Challenges in Achieving Realistic and Predictive Simulations of the Properties of Organic and Biological Molecules. Acc. Chem. Res. 1996, 29, 461–469. 10.1021/ar9500675.

[ref63] Gowers R.; Linke M.; Barnoud J.; Reddy T.; Melo M.; Seyler S.; Domański J.; Dotson D.; Buchoux S.; Kenney I.; Beckstein O. MDAnalysis: A Python Package for the Rapid Analysis of Molecular Dynamics Simulations. Proceedings of the 15th Python in Science Conference; U.S. Department of Energy, 2016; pp 98–105, 10.25080/majora-629e541a-00e.

[ref64] Michaud-Agrawal N.; Denning E. J.; Woolf T. B.; Beckstein O. MDAnalysis: A toolkit for the analysis of molecular dynamics simulations. J. Comput. Chem. 2011, 32, 2319–2327. 10.1002/jcc.21787. 21500218 PMC3144279

[ref65] Henderson L. J. CONCERNING, THE RELATIONSHIP BETWEEN THE STRENGTH OF ACIDS AND THEIR CAPACITY TO PRESERVE NEUTRALITY. Am. J. Physiol.-Leg. Content 1908, 21, 173–179. 10.1152/ajplegacy.1908.21.2.173.

[ref66] Vallat R. Pingouin: statistics in Python. J. Open Source Softw. 2018, 3, 1026 10.21105/joss.01026.

[ref67] Lundberg S.; Lee S.-I. A Unified Approach to Interpreting Model Predictions. arXiv 2017, 1705.07874 10.48550/arxiv.1705.07874.

[ref68] Arrow K. J.; Contributions to the Theory of Games (AM-28), Volume II; Princeton University Press, 1953; pp 307–318, 10.1515/9781400881970-018.

[ref69] Lundberg S. M.; Erion G.; Chen H.; DeGrave A.; Prutkin J. M.; Nair B.; Katz R.; Himmelfarb J.; Bansal N.; Lee S.-I. From local explanations to global understanding with explainable AI for trees. Nat. Mach. Intell. 2020, 2, 56–67. 10.1038/s42256-019-0138-9. 32607472 PMC7326367

[ref70] Eertink J. J.; Heymans M. W.; Zwezerijnen G. J. C.; Zijlstra J. M.; de Vet H. C. W.; Boellaard R. External validation: a simulation study to compare cross-validation versus holdout or external testing to assess the performance of clinical prediction models using PET data from DLBCL patients. EJNMMI Res. 2022, 12, 58 10.1186/s13550-022-00931-w. 36089634 PMC9464671

[ref71] Pedregosa F.; Varoquaux G.; Gramfort A.; Michel V.; Thirion B.; Grisel O.; Blondel M.; Müller A.; Nothman J.; Louppe G.; Prettenhofer P.; Weiss R.; Dubourg V.; Vanderplas J.; Passos A.; Cournapeau D.; Brucher M.; Perrot M.; Duchesnay É. Scikit-learn: Machine Learning in Python. arXiv 2012, 1201.0490 10.48550/arxiv.1201.0490.

[ref72] Ebo J. S.; Guthertz N.; Radford S. E.; Brockwell D. J. Using protein engineering to understand and modulate aggregation. Curr. Opin. Struct. Biol. 2020, 60, 157–166. 10.1016/j.sbi.2020.01.005. 32087409 PMC7132541

[ref73] Zhang C.; Samad M.; Yu H.; Chakroun N.; Hilton D.; Dalby P. A. Computational Design To Reduce Conformational Flexibility and Aggregation Rates of an Antibody Fab Fragment. Mol. Pharmaceut 2018, 15, 3079–3092. 10.1021/acs.molpharmaceut.8b00186. 29897777

[ref74] Codina N.; Hilton D.; Zhang C.; Chakroun N.; Ahmad S. S.; Perkins S. J.; Dalby P. A. An Expanded Conformation of an Antibody Fab Region by X-Ray Scattering, Molecular Dynamics, and smFRET Identifies an Aggregation Mechanism. J. Mol. Biol. 2019, 431, 1409–1425. 10.1016/j.jmb.2019.02.009. 30776431

