# Supplementary material for: Predictive Model Building for Aggregation Kinetics Based on Molecular Dynamics Simulations of an Antibody Fragment
Source: Mol Pharm. 2024 Sep 30;21(11):5827–41. doi: 10.1021/acs.molpharmaceut.4c00859 (PMC11539058; doi:10.1021/acs.molpharmaceut.4c00859)
Supplement: Supplementary file 1 — mp4c00859_si_001.pdf [file mp4c00859_si_001.pdf]

Supplementary Information:

Predictive model building for aggregation kinetics based on molecular dynamics simulations of an antibody fragment

Yuhan Wang[1], Hywel D Williams[2], Duygu Dikicioglu[1], Paul A Dalby[1]

1. Department of Biochemical Engineering, University College London, London, WC1E 6BT, UK

2. CSL Ltd, Biopharmaceutical Product Development, 45 Poplar Road, Parkville, 3052 Australia

Fab A33 sequence information

Fab A33 amino acid sequence separated by domains. The six CDRs in the V_L and V_H domains are highlighted in red.

V_L
DIQMTQSPSSLSASVGDRVTITCKASQNVRTVVAWYQQKPGKAPKTLIYLASNRHTGVPS
RFSGSGSGTDFTLTISSLQPEDFATYFCLQHWSYPLTFGQGTKVEIKR

C_L
TVAAPSVFIFPPSDEQLKSGTASVVCLLNNFYPREAKVQWKVDNALQSGNSQESVTEQD
SKDSTYSLSSTLTLSKADYEKHKVYACEVTHQGLSSPVTKSFNRGEC

V_H
EVQLVESGGGLVQPGGSLRLSCAASGFAFSTYDMSWVRQAPGKGLEWVATISSGGSYT
YYLDSVKGRFTISRDSSKNTLYLQMNSLRAEDTAVYYCAPTTVVPFAYWGQGTLVTVSS
AST

C_H1
KGPSVFPLAPSSKSTSGGTAALGCLVKDYFPEPVTVSWNSGALTSGVHTFPAVLQSSGL
YSLSSVVTVPSSSLGTQTYICNVNHKPSNTKVDKKV

Hinge
EPKSCDKTHTSAA

A benchmark of different force fields of all-atom simulations

The benchmark study investigated the difference between four Gromacs built-in force fields: OPLS, Amber 94, Amber 99SB, Amber 96. It showed no significant difference between the four force fields after conducting 10 ns simulations on Fab A33 for each force field, six replicas for each.

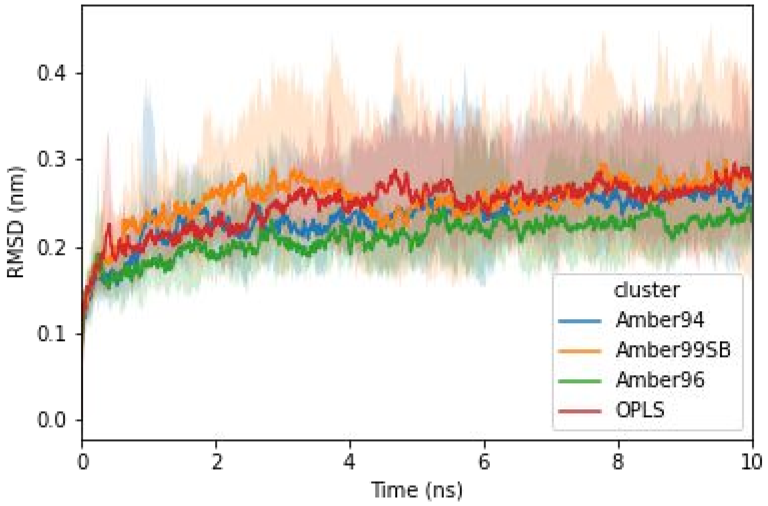

Figure S1. The comparison of the RMSD between four different built-in force fields in Gromacs software, Amber 94, Amber 99SB, Amber 96, OPLS. Six replicas were run for each force field for 10 ns. Four different colours indicated different force fields as labelled in the chart. Solid lines show the average RMSD from the six replicas, and the background outlines show the ranges (minimum to maximum) of the RMSD values over time.

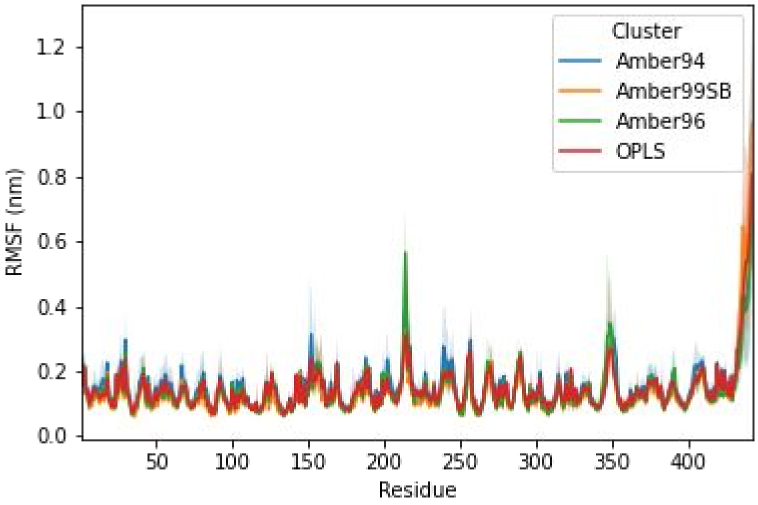

Figure S2. The comparison of the RMSF between four different built-in force fields in Gromacs software, Amber 94, Amber 99SB, Amber 96, OPLS. Six replicas were run for each force field for 10 ns. Four different colours indicated different force fields as labelled in the chart. Solid lines show the average RMSF from the six replicas, and the background outlines show the ranges (minimum to maximum) of the RMSF values over time.

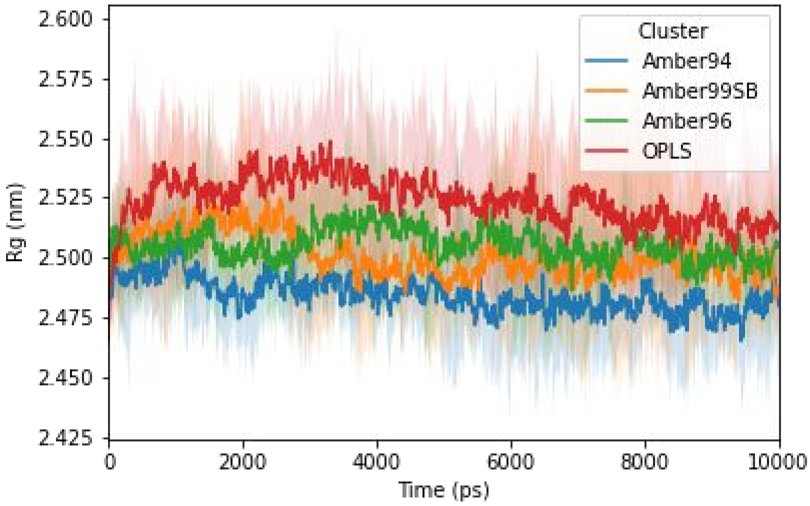

Figure S3. Comparison of the radius of gyration (R_g) between four different built-in force fields in Gromacs software, Amber 94, Amber 99SB, Amber 96, OPLS. Six replicas were run for each force field for 10 ns. Four different colours indicated different force fields as labelled in the chart. Solid lines show the average R_g for the six replicas, and the background outlines show the ranges (minimum to maximum) of the R_g values over time.

Principal component analysis on the 49 condition dataset

To investigate the contribution of each of the three variables, temperature, pH, and ionic strength (IS) to the experimental aggregation kinetics, a principal component analysis was conducted to calculate the principal components that can explain most of the data. The covariance matrix was calculated from the scaled dataset and eigenvalues and eigenvectors from the covariance matrix were obtained. A loading plot that shows variables represented in the PC space was generated. The number of the PCA components was set up as 3 in this analysis.

The first principal component explained 46% of the whole dataset while the second principal component explained 32%, contributing a total of 78%. IS and pH were mainly represented by PC1, while temperature was equally represented by PC1 and PC2.

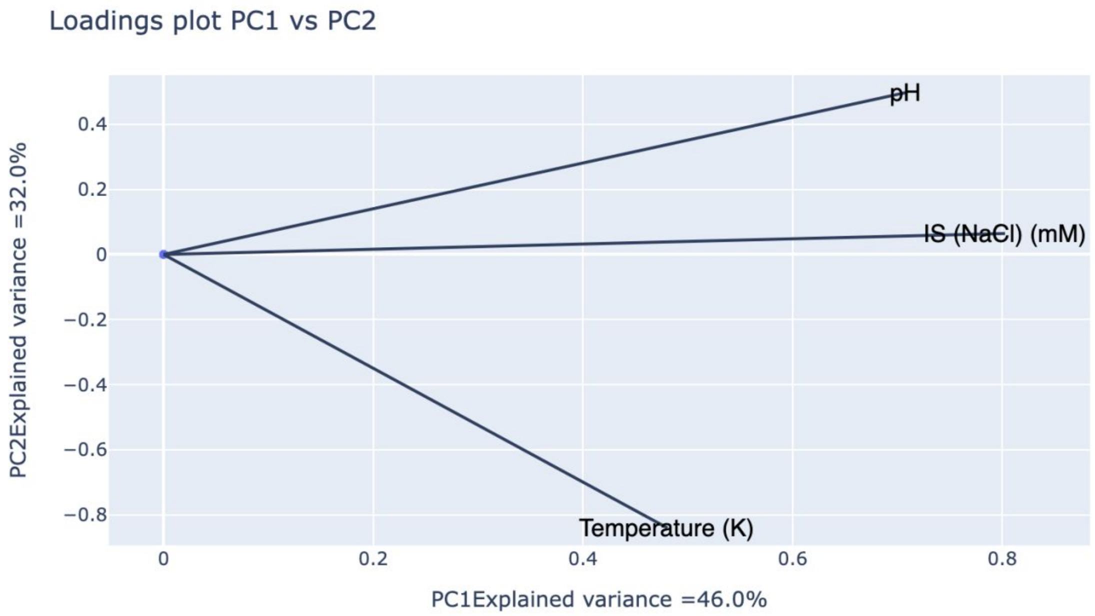

Figure S4. Loading plot from the PCA. This shows the relationship between the three variables, temperature, pH, and ionic strength, and how they are represented by principal components.

Plots of RMSD, RMSF and radius of gyration over 100ns for 49 conditions with 6 replicas each

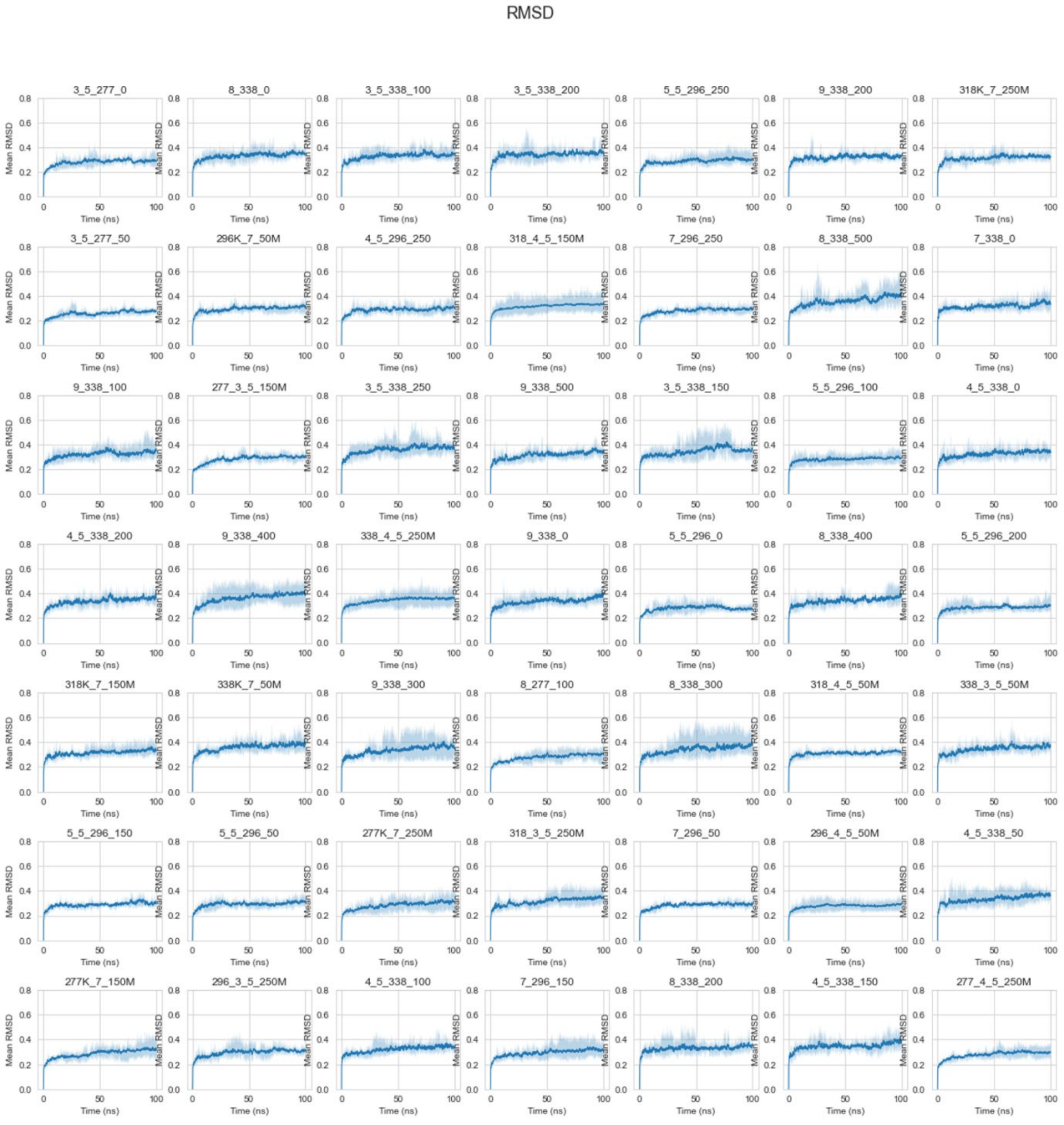

Figure S5. RMSD over time in 49 conditions. Solid blue lines show the average RMSD across six replicas whereas the shadow area is the minimum and maximum values in the six replicas. Each subplot has the condition as its title.

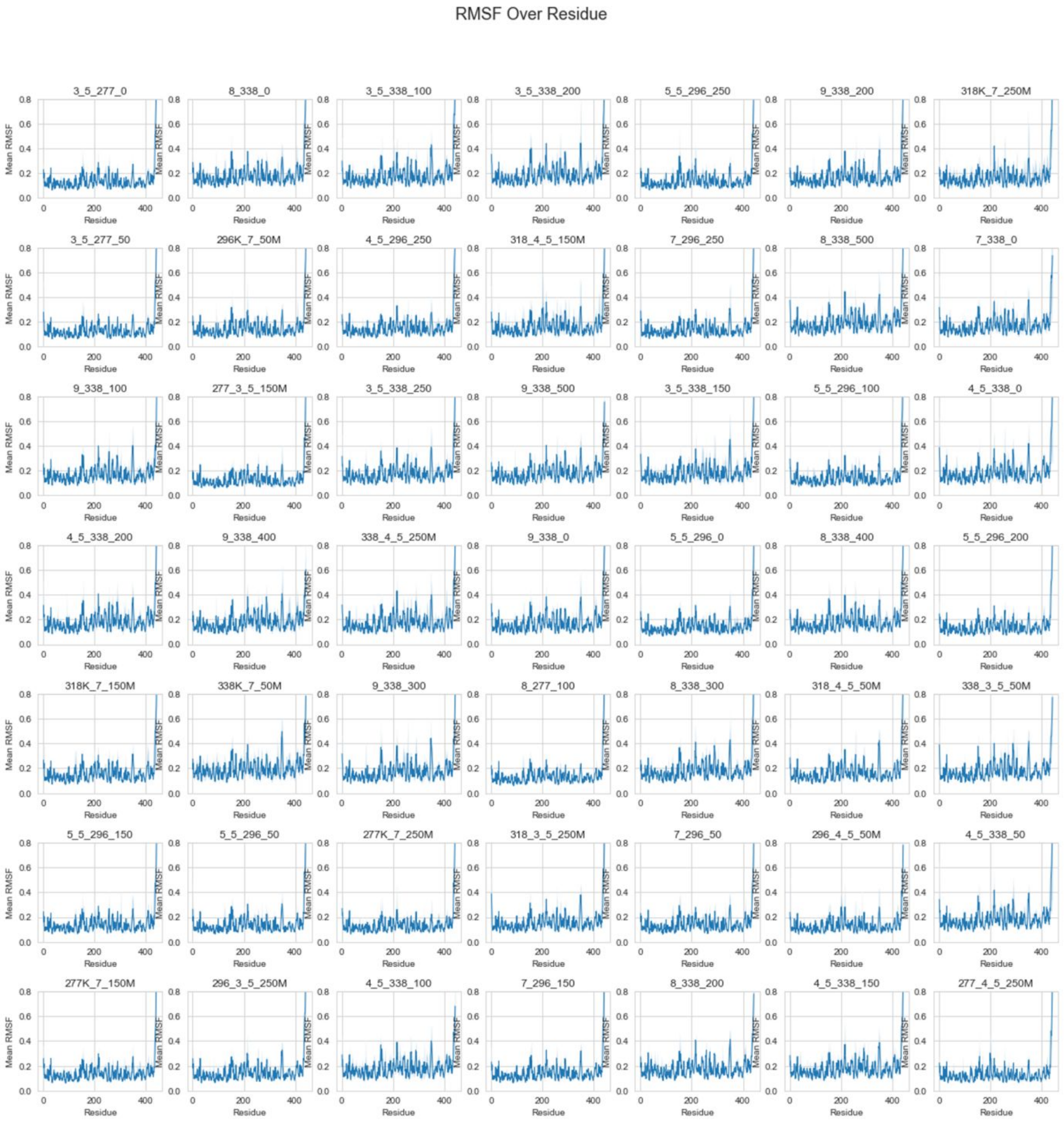

Figure S6. RMSF over time in 49 conditions. Solid blue lines show the average RMSF across six replicas whereas the shadow area is the minimum and maximum values in the six replicas. Each subplot has the condition as its title.

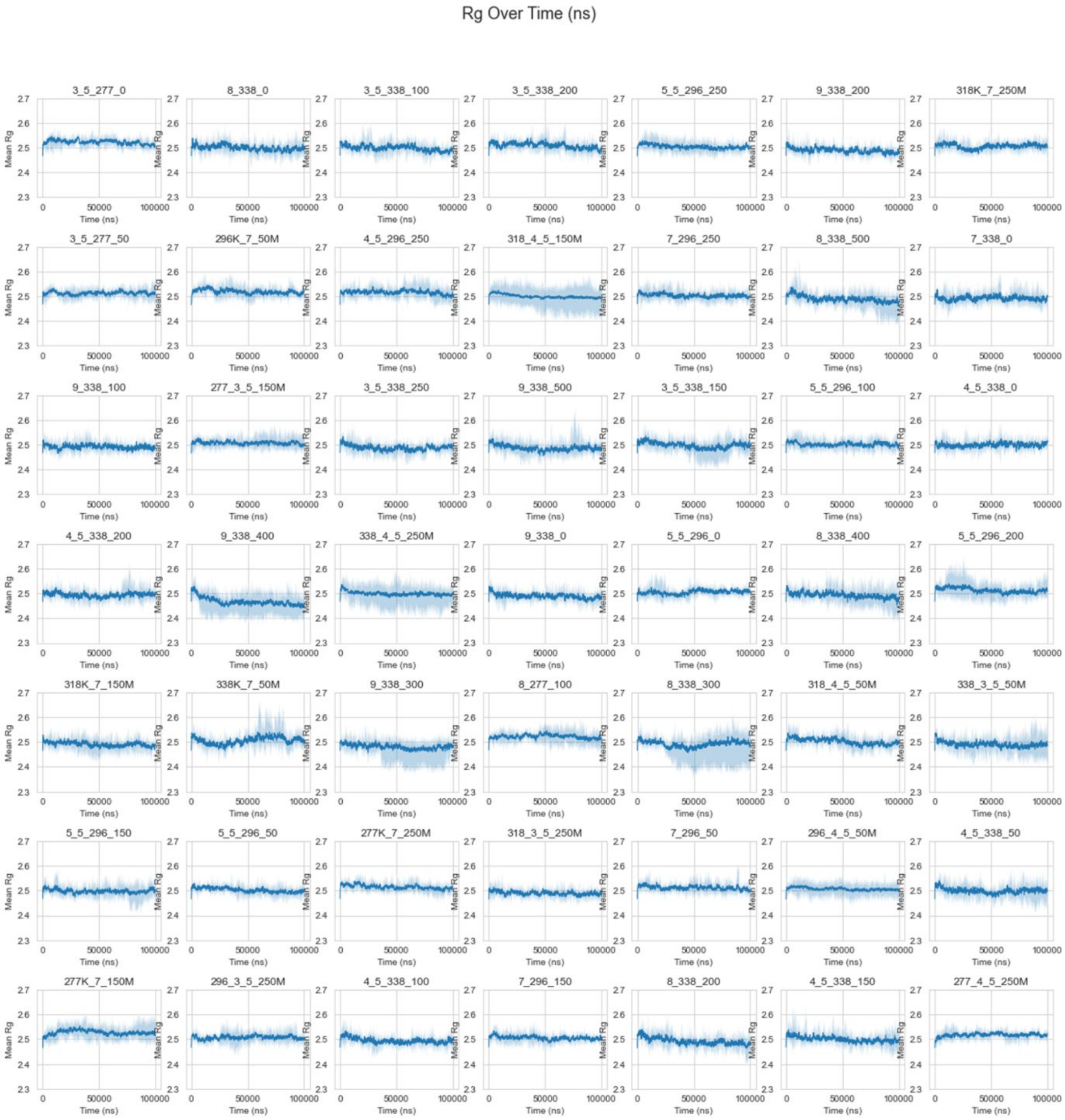

Figure S7. Radius of gyration (R_g) over time in 49 conditions. Solid blue lines show the average R_g across six replicas whereas the shadow area is the minimum and maximum values in the six replicas. Each subplot has the condition as its title.

The distribution analysis of RMSD and radius of gyration (R_g) at four different temperatures

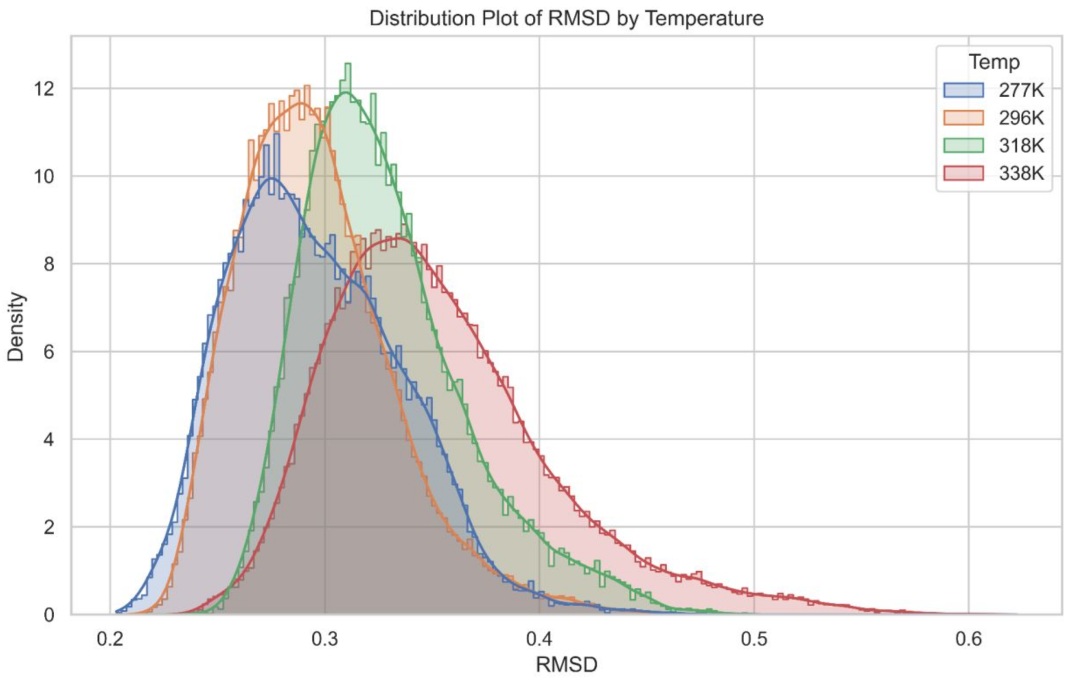

Figure S8. A normalised histogram with KDE lines showing the distributions of RMSD at four different temperatures, 277K, 296K, 318K, 338K.

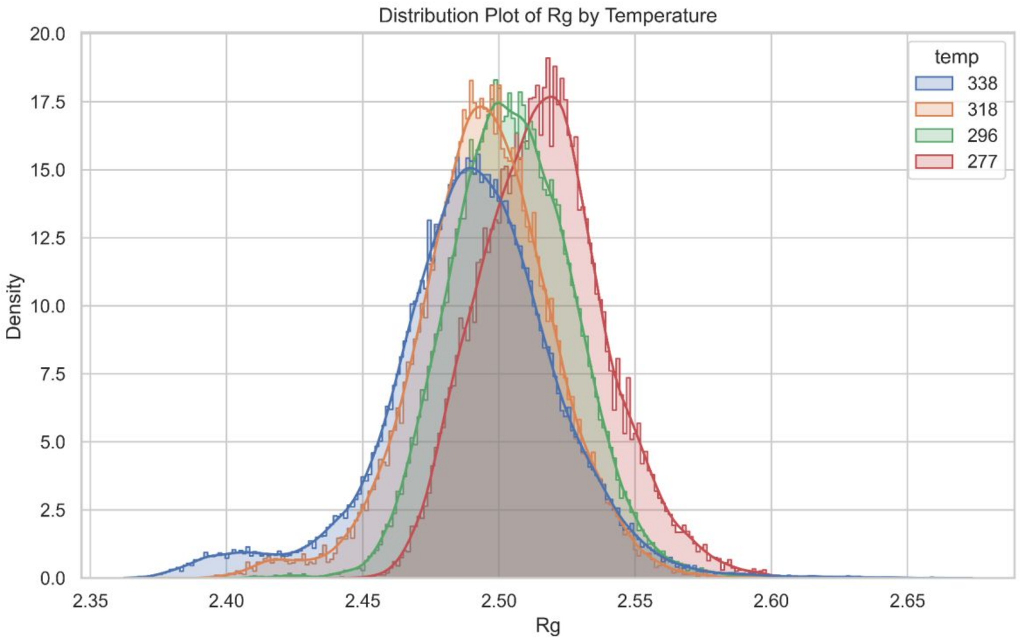

Figure S9. A normalised histogram with KDE lines showing the distributions of radius of gyration (R_g) at four different temperatures, 277K, 296K, 318K, 338K.

The residue-level RMSF over a range of selected conditions

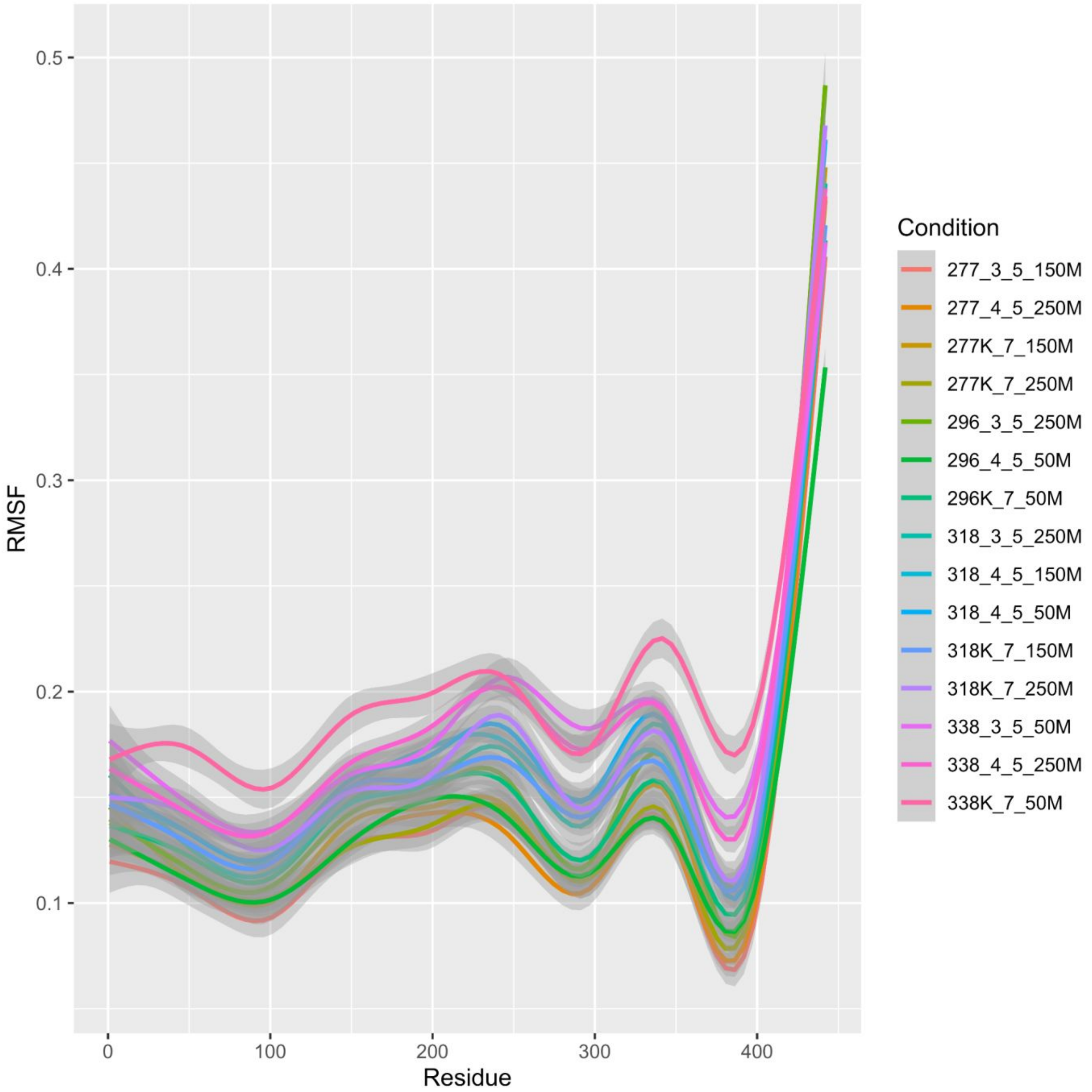

Figure S10. A distribution plot of average RMSF at fifteen selected conditions. The coloured lines are the average RMSF across six replicas and the shadows are the maximum and minimum RMSF values across the six replicas. Only 15 conditions were shown in order to retain clarity in the Figure.

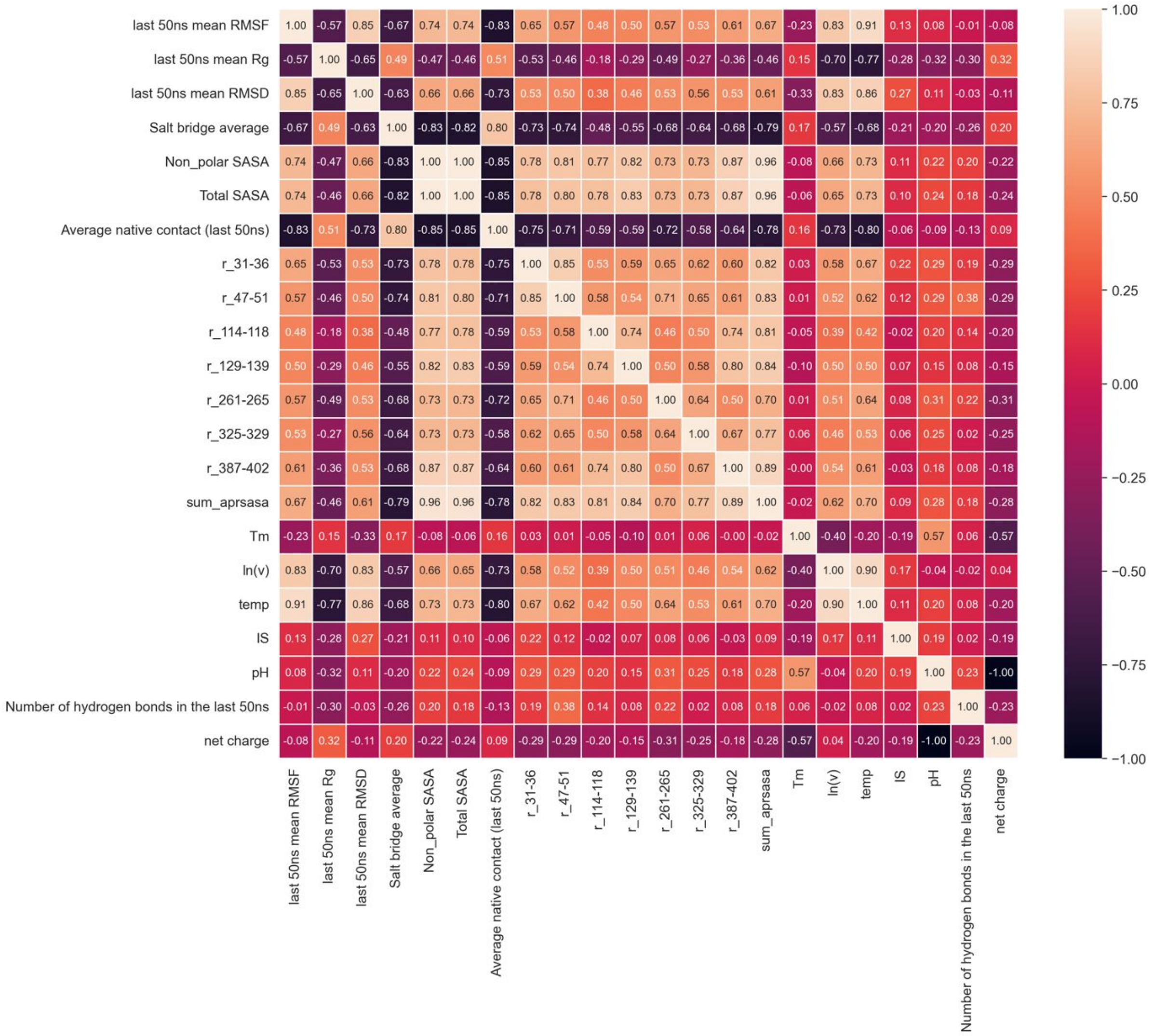

Figure S11. Spearman correlation matrix. A heatmap of Spearman correlations (as r values) between all the variable features, experimental variables (as labels during the model building), and experimental data of melting temperature (T_m) and aggregation kinetics (ln(v)). The colour code shown on the right side with black indicates a strong negative correlation whereas white or mild orange indicates a strong positive correlation. Non-polar SASA: solvent accessible surface area for all the carbon atoms in the protein; Total SASA: solvent accessible surface area for all the atoms except for H in the protein; average native contacts (last 50ns): the average fraction of native contacts derived from the last 500 frames; r_31-36, r_47-51, r_114-118, r_129-139, r_261-265, r_325-329, r_387-402: the SASA of the 7 APR regions on Fab A33, respectively; sum_aprsasa: sum of the ΔSASA of the 7 APRs on Fab A33; mean RMSF: global average RMSF across six replicas; last 20 ns mean Rg: the last 20 ns average radius of gyration across six replicas; last 20 ns mean RMSD: the last 20 ns average RMSD across six replicas; number of hydrogen bonds in the last 50 ns: the number of hydrogen bonds averaged over the last 50 ns across six independent replicas; net charge: the total net charge of the protein at different pHs

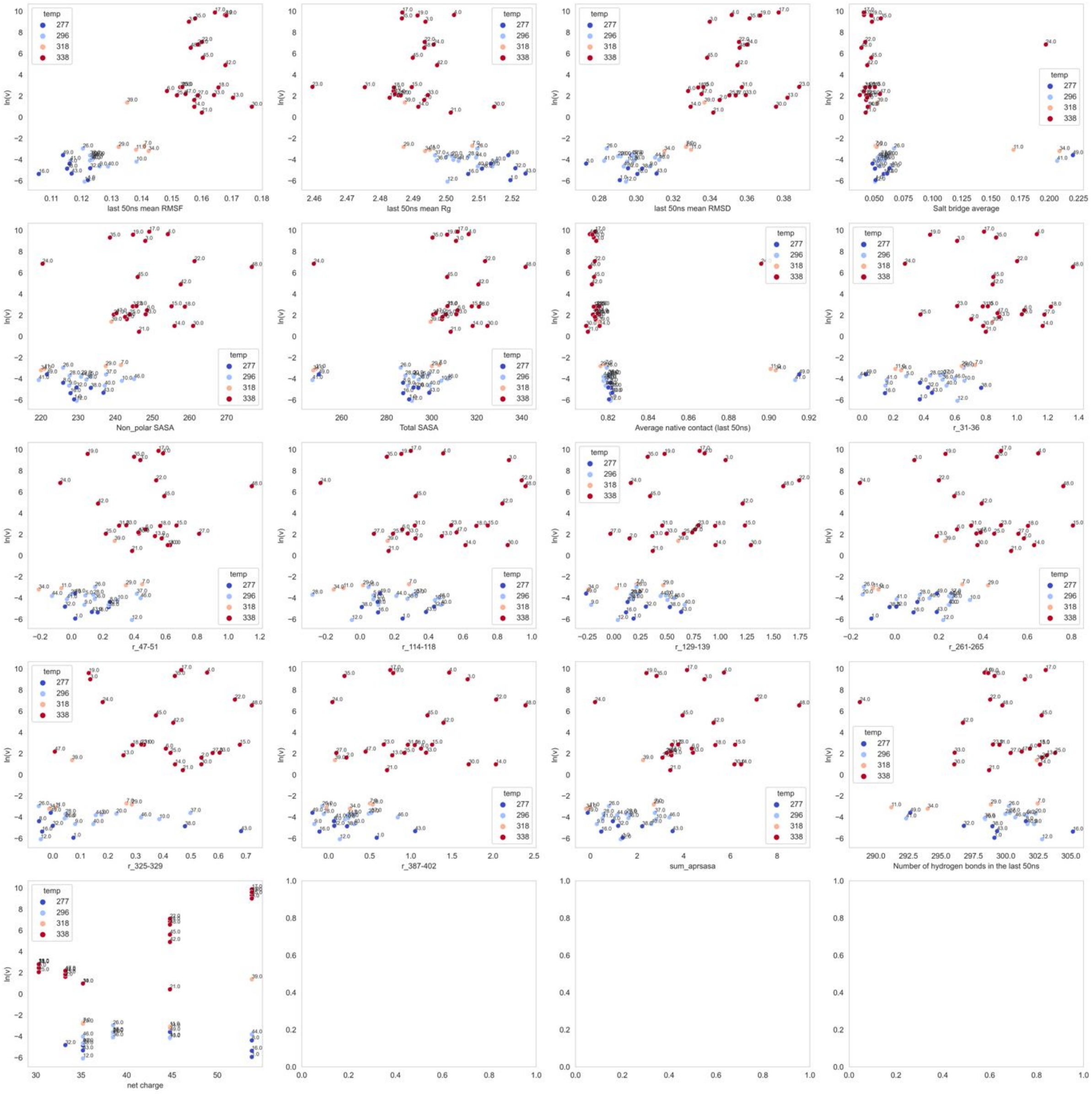

Figure S12. Scatter plots with each molecular feature values as X axis and experimental aggregation kinetics as Y axis. Experimental temperatures are colour coded.

Model building for predicting aggregation kinetics

Table S1. Comparing the performance of five regression models with 10-fold cross validation and leave-one-out cross-validation (LOOCV). The mean MSE, maximum MSE, standard deviation of MSE, and R^2, were calculated for each model.

**Model**	Ideal (LOOCV) MSE	10-fold cross validation MSE	R^2
Multiple linear regression (MLR)	5.898 ± 7.192 ((max=13.089))	5.583 ± 2.377 (max=7.961)	0.558
Partial linear analysis (PLS)	5.642 ± 7.411 (max=13.052)	5.812 ± 3.287 (max=9.099)	0.580
Support vector regression (SVR)	10.370 ± 20.278 (max=30.648)	10.587 ± 6.694 (max=17.281)	0.515
Decision tree regression	4.711 ± 14.511 (max=19.222)	5.215 ± 5.945 (max=11.159)	0.676
Random forest regression	3.897 ± 12.228 (max=16.125)	3.738 ± 4.760 (max=8.499)	0.759

Model building for predicting fraction of unfolding

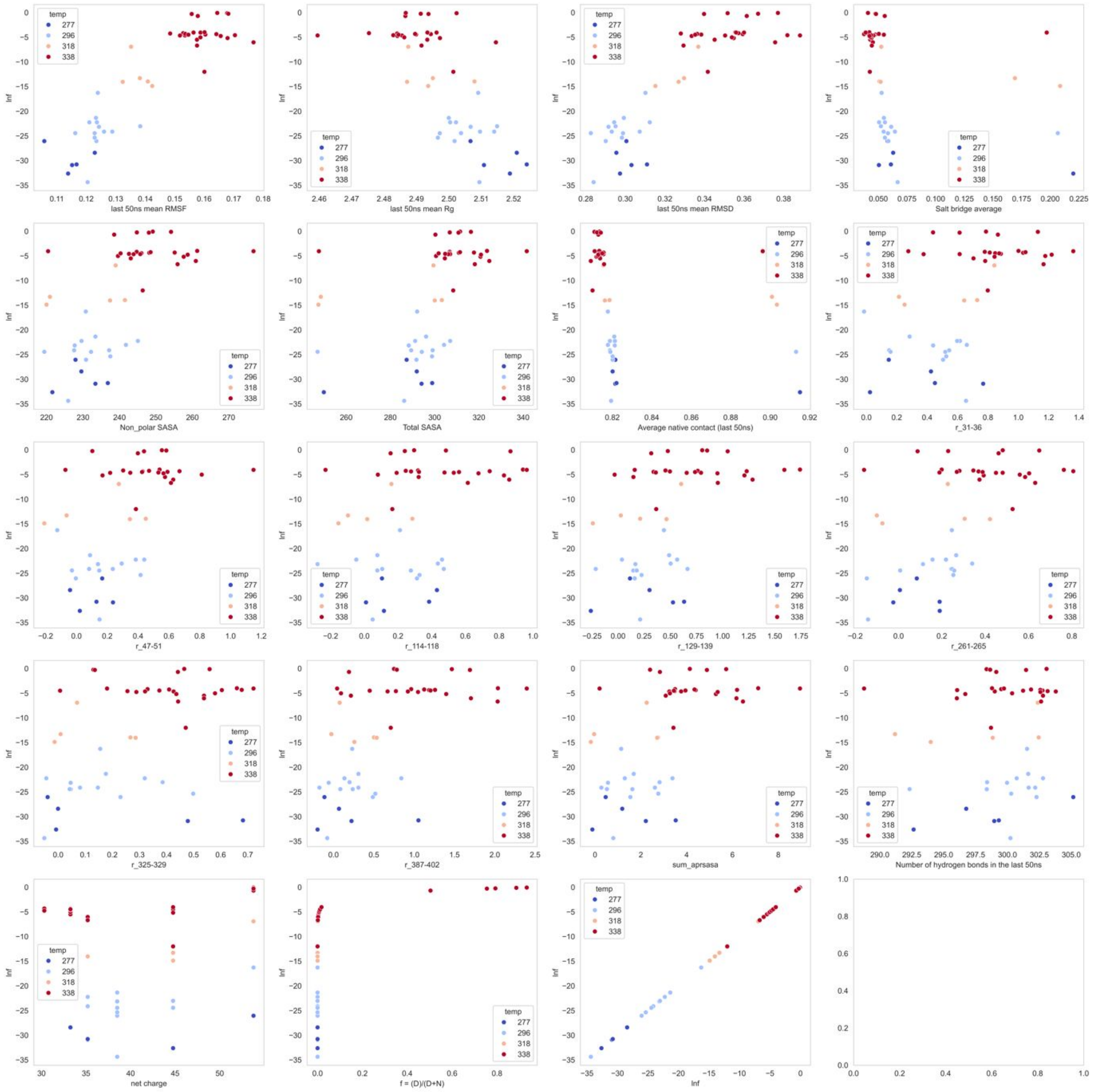

Figure S13. Scatter plots of 17 molecular features calculated from MD simulations versus the experimental natural log-value of fraction unfolding (ln(f)). Plots are colour-coded by temperature.

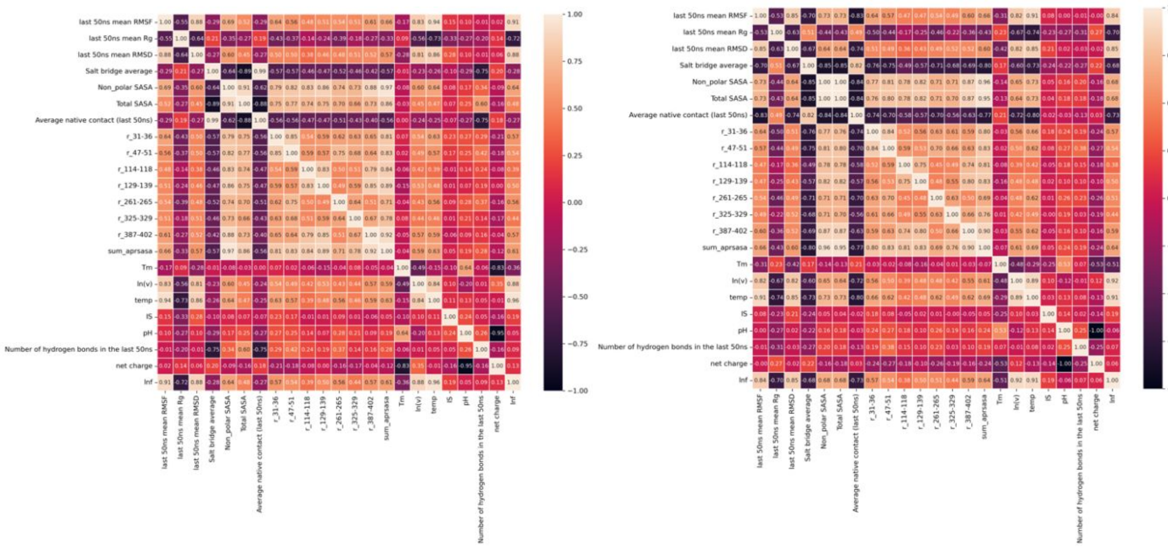

Figure S14. Pearson (LHS) and Spearman (RHS) correlations between features, aggregation kinetics, and fraction of unfolding.

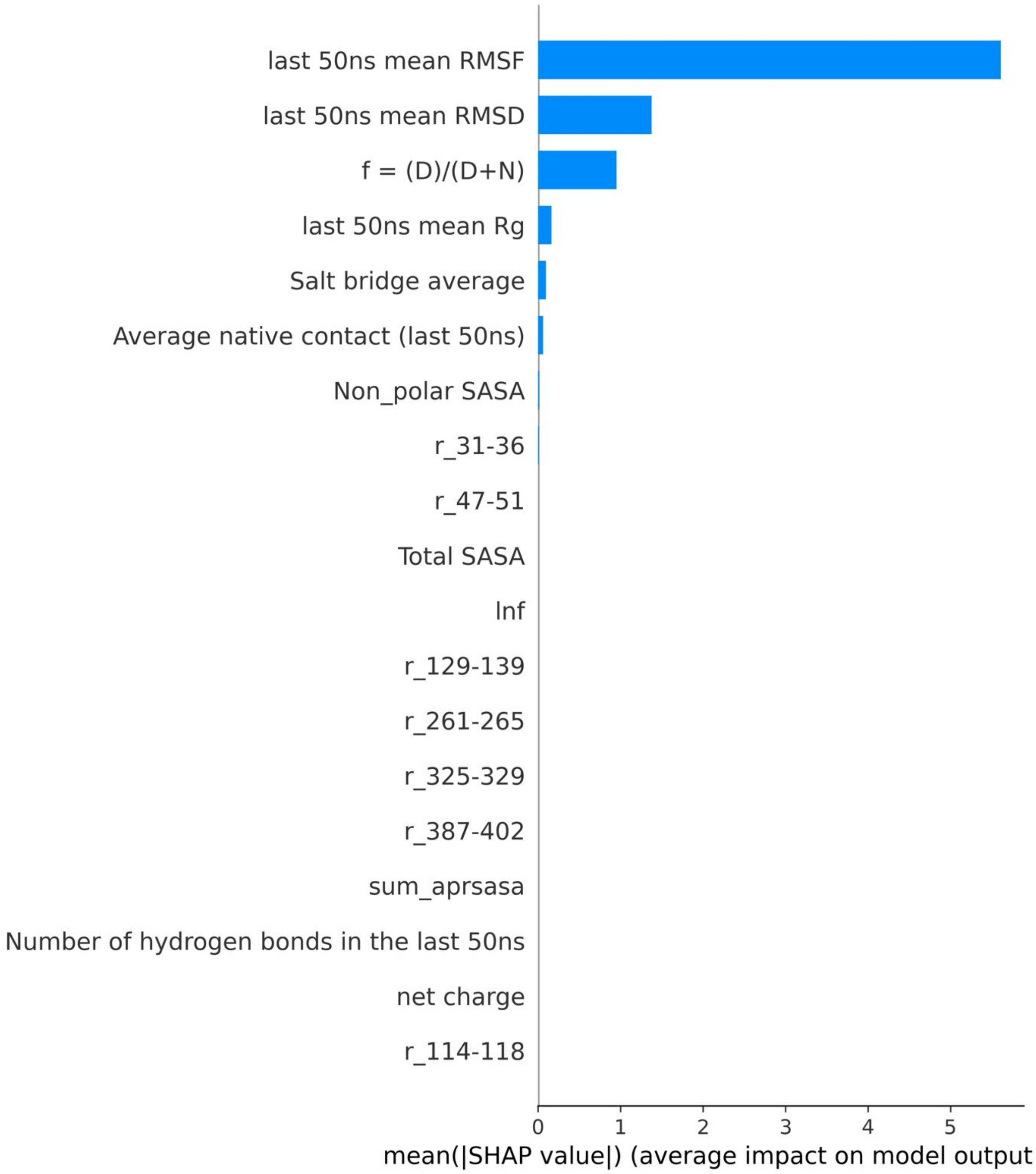

Figure S15. A depth 1 model trained with xgboost.XGBRegressor using SHAP method. It ranked the contribution of each feature to the model buidling.

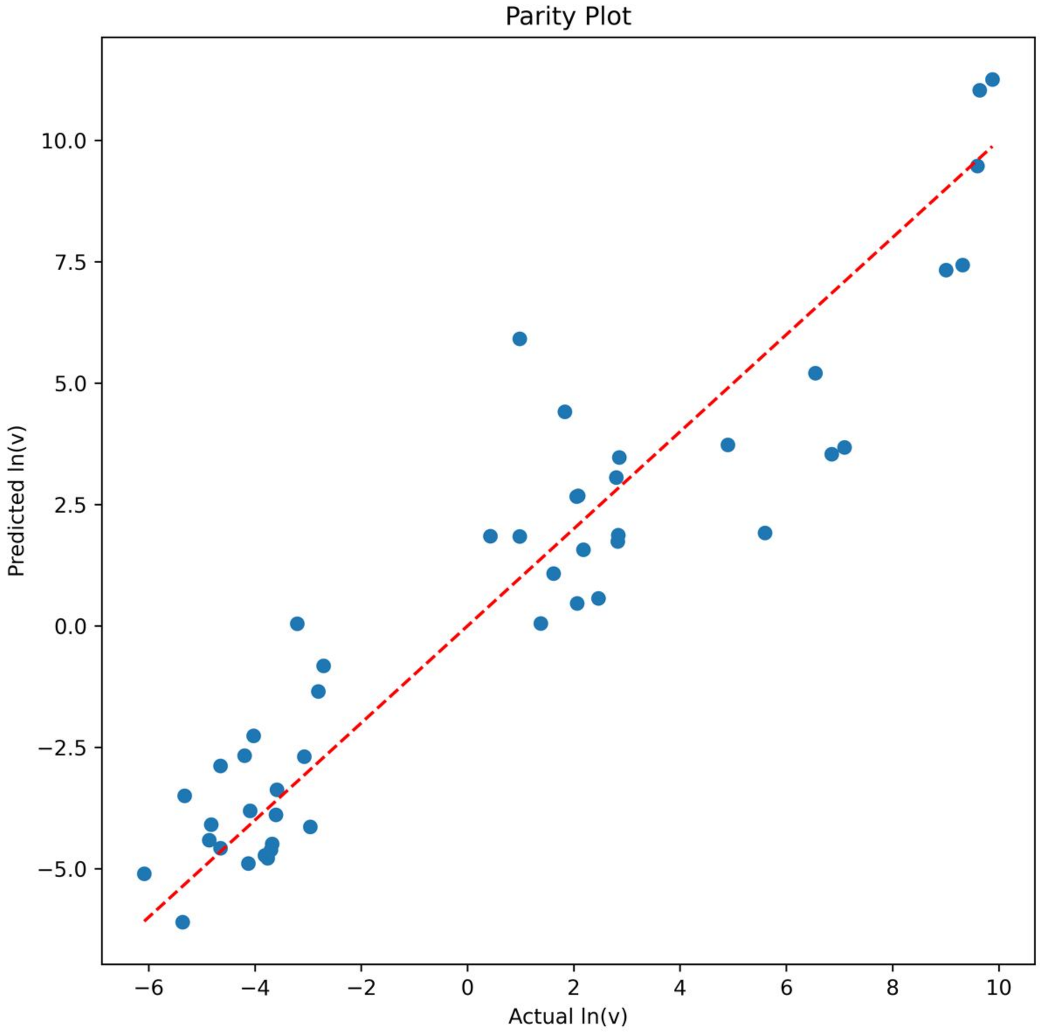

Figure S16. A multiple linear regression model built for predicting fraction of unfolding with an R^2 of 0.88 trained on the whole dataset after evaluating different models with cross validation. It had the best performance tested among all the models.
